# Identification of the gene signature reflecting schizophrenia’s etiology by constructing artificial intelligence‐based method of enhanced reproducibility

**DOI:** 10.1111/cns.13196

**Published:** 2019-07-27

**Authors:** Qing‐Xia Yang, Yun‐Xia Wang, Feng‐Cheng Li, Song Zhang, Yong‐Chao Luo, Yi Li, Jing Tang, Bo Li, Yu‐Zong Chen, Wei‐Wei Xue, Feng Zhu

**Affiliations:** ^1^ College of Pharmaceutical Sciences Zhejiang University Hangzhou China; ^2^ School of Pharmaceutical Sciences Chongqing University Chongqing China; ^3^ Bioinformatics and Drug Design Group, Department of Pharmacy National University of Singapore Singapore Singapore

**Keywords:** reproducibility, schizophrenia, significant analysis of microarray, student's *t* test, transcriptomics

## Abstract

**Aims:**

As one of the most fundamental questions in modern science, “what causes schizophrenia (SZ)” remains a profound mystery due to the absence of objective gene markers. The reproducibility of the gene signatures identified by independent studies is found to be extremely low due to the incapability of available feature selection methods and the lack of measurement on validating signatures’ robustness. These irreproducible results have significantly limited our understanding of the etiology of SZ.

**Methods:**

In this study, a new feature selection strategy was developed, and a comprehensive analysis was then conducted to ensure a reliable signature discovery. Particularly, the new strategy (a) combined multiple randomized sampling with consensus scoring and (b) assessed gene ranking consistency among different datasets, and a comprehensive analysis among nine independent studies was conducted.

**Results:**

Based on a first‐ever evaluation of methods’ reproducibility that was cross‐validated by nine independent studies, the newly developed strategy was found to be superior to the traditional ones. As a result, 33 genes were consistently identified from multiple datasets by the new strategy as differentially expressed, which might facilitate our understanding of the mechanism underlying the etiology of SZ.

**Conclusion:**

A new strategy capable of enhancing the reproducibility of feature selection in current SZ research was successfully constructed and validated. A group of candidate genes identified in this study should be considered as great potential for revealing the etiology of SZ.

## 1 INTRODUCTION

Schizophrenia (SZ) is severe and chronic disorder characterized by abnormal interpretations of reality.^1^, ^2^, ^3^ It affects over one percent of the global population^4^ and brings about the extremely distorted psychological and physiological behaviors with the reduction of life expectancy by 20 years compared with that of healthy individuals.^5^ As one of the most fundamental questions in modern science,^6^ “what causes SZ” remains a profound mystery due to the absence of objective molecular markers.^7^, ^8^, ^9^, ^10^ To answer this, the discovery of the essential genes in SZ’s occurrence/development has been extensively explored,^11^, ^12^, ^13^ and the microarray screening combining the filter‐based feature selection approaches (such as Student's *t* test and significant analysis of microarray) has emerged as one of the most effective tools.^14^, ^15^, ^16^, ^17^, ^18^, ^19^ Using this tool, some genes of differential expression (DEGs, like *S100A8* 20) between patients with SZ and healthy individuals are identified, and the pathways susceptible to SZ (including neurotrophin signaling^21^, ^22^, ^23^) or vital in SZ‐induced cognitive impairment (including natural killer mediated cytotoxicity^24^, ^25^, ^26^) are discovered.

However, the sets of disease‐related DEGs discovered from different microarray experiments are reported to be largely irreproducible,^27^ and the analytical results of the previous studies on SZ gene expression vary significantly.^14^, ^28^ Particularly, no gene is simultaneously top‐ranked by seven separate studies as the DEG in SZ.^29^ This irreproducibility can significantly hamper the reliability of the identified disease signature^30^ and restrict the clinical application of the discovered DEGs.^8^ Moreover, this may be the reason why there is no objective molecular marker available for the diagnosis or treatment of SZ, and why the cause of SZ remains a profound mystery in the community of biomedical researches.^31^

The irreproducibility among the signatures discovered from independent studies has been attributed to (a) the limited abilities of available feature selection approaches to detect the sophisticated/subtle changes in SZ gene expression^14^, ^32^ and (b) the lack of effective measurements on validating the robustness of analytic results.^20^, ^29^ In recent year, some wrapper or embedded approaches for selecting features were reported to outperform the traditional filter ones.^33^, ^34^, ^35^, ^36^ However, these available approaches did not effectively take the robustness among different signatures discovered by independent studies into consideration,^27^, ^37^ and it is thus crucial to construct novel approaches with significantly enhanced reproducibility. Moreover, the analytical methodology together with multiple randomized sampling is known as capable of validating the robustness of analytic results and usefulness of identified markers by drawing conclusion over multiple independent studies.^37^, ^38^, ^39^, ^40^, ^41^ Due to its ability to produce the more comprehensive and broader conclusions than traditional measurements,^42^ the comprehensive analyses have been applied to empirically investigate the replicability failure in current SZ research,^43^, ^44^ substantially facilitate the discovery of risk allele^45^ or gene marker^46^, ^47^, ^48^ of SZ, and systematically assess the drug response rate of patient with SZ.^49^, ^50^, ^51^, ^52^ It is therefore of great interest in constructing novel feature selection strategy of significantly enhanced reproducibility.

Herein, a comprehensive analysis of datasets from multiple independent microarray studies was conducted, and a novel feature selection strategy was developed to ensure the reliable signature discovery. As assessed, the SZ gene signature identified using this strategy was highly accurate and reproducible. A forest plot of the results of independent test datasets was applied to evaluate the level of enhancement in reproducibility of this strategy compared with the traditional ones that were adopted in SZ research. In sum, these findings not only confirmed the successful construction of a novel feature selection strategy capable of enhancing the discovery robustness of SZ molecular signature but also facilitated the identification of candidate genes in revealing the molecular mechanism underlying the cognitive dysfunction in patients with SZ.

## 2 MATERIALS AND METHODS

### 2.1 Selection of independent studies and data preprocessing

A variety of public databases providing microarray data were comprehensively reviewed. These databases included Gene Expression Omnibus (GEO, http://www.ncbi.nlm.nih.gov/geo/), Stanley Medical Research Institute Online Genomics Database (SMRI),^16^ and Harvard Brain Bank database (HBB).^14^ The GEO was systematically searched using such keyword as “schizophrenia”, “schizophrenia patients”, “schizophrenia subjects”, “schizophrenic patients” and “schizophrenic subjects”, which gave the microarray datasets with one cohort of SZ subjects and another cohort of healthy people. All datasets in SMRI database were batch downloaded and processed to assess whether they included both patients and controls. The corresponding data from the HBB database were collected from published study.^14^ In addition to these three popular SZ‐related databases, the systematic literature reviews based on the libraries of PubMed, PsycINFO, Embase, and Cochrane were also conducted using the same keywords as provided above. For the resulting datasets, their affiliated information, including the organism/species origins (human, mouse, HPV etc), study types (expression profiling, genome variations, methylation, noncoding RNAs, proteins etc), and specific brain loci (frontal cortex, hippocampus, caudate nucleus etc), was extensively collected for analysis. Duplicates among those resulting datasets were systematically removed.

Based on the information affiliated to each dataset, the collected studies were further selected if they met the following inclusion criteria: (a) gene expression profiling based on cDNA microarray technology; (b) tissues collected from prefrontal cortex (PFC, defined as *Brodmann areas* BA9, BA10 & BA46, since PFC has been widely accepted as the major locus of SZ dysfunction based on the results from both clinical and neuroimaging studies); (c) the raw dataset (CEL file) available for analysis; (d) consisted of one cohort of patients and another cohort of healthy controls; and (5) species origin of “*Homo Sapiens.*” As demonstrated in Table S1, the searching process/history, screening, and dataset inclusion in each electronic database were described. *First*, the numbers of resulting records by direct keyword searches in the libraries of GEO, SMRI, HBB, PubMed, PsycINFO, Embase, and Cochrane were 4,256, 20, 1, 505, 942, 1,346, and 13, respectively. *Then*, the numbers of resulting records by following the above 5 sequential criteria were provided in Table S1. *Third*, the numbers of datasets passing all 5 sequential criteria for the libraries of GEO, SMRI, HBB, PubMed, PsycINFO, Embase, and Cochrane were 4, 2, 1, 9, 0, 8, and 0, respectively. *Finally*, nine independent microarray studies were collected and included in this analysis by removing the duplicates across all electronic database. As shown in Table 1, each independent study was assigned a unique ID (from A to I). Specifically, 5, 3, and 1 studies were conducted using the platforms of HG‐U133A, HG‐U133 Plus 2, and Agilent‐014850, respectively, and 5, 2, 1, and 1 studies focused on the brain regions of BA46, BA10/46, BA9, and BA10, respectively. Moreover, the sample sizes of these studies varied substantially (from the 15 to 65), and 4, 2, 1, and 2 studies had the sample sizes of >50, 40‐50, 30‐40, and ≤30, respectively. All analyses reported here were conducted in the *R* environment (v3.4.3). The raw data (CEL file) were read, log‐transformed, and normalized using the *R* package of *affy* and *limma,* and the parameters were all set to default. Then, probe sets were mapped to their corresponding genes, and the average expression value was retained if one gene was mapped to multiple probes.

**Table 1:** Datasets collected from nine independent microarray studies (sorted by sample size)

ID	Dataset reference	Brodmann's area code	Sample size (SZ:HEA)	Platform ID	Platform description
A	*BMC Genomics*. 7:70, 2006	46	65 (34:31)	GPL96	Affymetrix Human Genome U133A Array (HG‐U133A)
B	*Schizophr Res*. 77:241‐252, 2005	10/46	60 (31:29)	GPL96	Affymetrix Human Genome U133A Array (HG‐U133A)
C	*Schizophr Res*. 161:215‐221, 2015	46	59 (29:30)	GPL4133	Whole Human Genome Microarray 4x44K G4112F (Agilent‐014850)
D	*Brain Res*. 1239:235‐248, 2008	46	54 (25:29)	GPL570	Affymetrix Human Genome U133 Plus 2.0 Array (HG‐U133 Plus 2)
E	*Mol Psychiatry*. 14:1083‐1094, 2009	10	47 (26:21)	GPL570	Affymetrix Human Genome U133 Plus 2.0 Array (HG‐U133 Plus 2)
F	*Proc Natl Acad Sci USA*. 102:15533‐15538, 2005	9	45 (19:26)	GPL96	Affymetrix Human Genome U133A Array (HG‐U133A)
G	*PLoS One*. 10:e0121744, 2015	46	32 (13:19)	GPL570	Affymetrix Human Genome U133 Plus 2.0 Array (HG‐U133 Plus 2)
H	*BMC Psychiatry*. 8:87, 2008	10/46	20 (09:11)	GPL96	Affymetrix Human Genome U133A Array (HG‐U133A)
I	*Neuropsychopharmacol H*. 10:9‐14, 2008	46	15 (09:06)	GPL96	Affymetrix Human Genome U133A Array (HG‐U133A)

Notes

These studies were in vivo investigations conducted within the prefrontal cortex of the postmortem brain tissue. Each dataset contained one cohort of SZ subjects (SZ) and another cohort of healthy individuals (HEA). The study IDs assigned in this table were used to indicate those nine datasets throughout the manuscript.

### 2.2 Consistent discovery of gene signature based on the newly constructed strategy

As one of the most popular machine learning algorithms, the *support vector machine* (SVM) showed good performance in classifying microarray datasets,^53^, ^54^ and the corresponding wrapper or embedded recursive feature elimination algorithm (SVM‐RFE^55^) was widely used in current study.^37^ During SVM‐RFE‐based feature selection, a gene ranking function was initially generated based on the *artificial intelligence* (AI) classifier (SVM), and the signature was then identified by discarding the genes that were not differentially expressed.^55^ In this study, a novel strategy based on SVM‐RFE was thus proposed and constructed by (a) integrating repeated random sampling with consensus scoring and (b) evaluating the consistency of gene rankings among multiple independent datasets. Workflow of this strategy was provided in Figure S1 and described in detail below.

First, one study (the *i*th study) was randomly selected from the nine independent studies, and the remaining eight studies were used as independent test datasets. The *i*th study was separated into 1000 unique training‐test datasets using repeated random sampling.^27^ For each training‐test dataset, half of the SZ patient cohort and half of the healthy cohort were randomly selected to construct the training dataset, and the remaining samples were all placed in the corresponding test dataset.

Then, the 1000 training‐test datasets were randomly grouped into 10 sampling groups (each of 100 unique training‐test datasets). In each dataset, the signature was identified from the training dataset by SVM‐RFE, and the corresponding test set was used to assess the classification performance of the identified signature. The consistency of gene rankings among 100 training‐test sets in each sampling group was assessed using the sequential methods of consensus scoring described below to enhance the consistency among signatures identified from different datasets: (α) the genes ranked in the bottom (<50%, depending on the number of remaining genes in different rounds) were selected out if their collective contribution did not surpass the top‐ranked genes; (β) among the selected genes, those ranked in the bottom half during previous round of ranking were chosen to ensure that they consistently received low rankings among iterations; and (γ) the low‐ranking genes appearing in >90% of the 100 training‐test datasets were discarded.

Finally, the signature was identified based on the highest average classification accuracy among 100 test datasets. Ten sampling groups were all analyzed using the same method, and the gene signature comprised DEGs that were simultaneously identified from all sampling groups. All calculations were achieved using a high‐performance computing (HPC) server with 768 GB RAM and CPU E7‐8168 × 24 cores and further accelerated by GPU NVIDIA Tesla K80. Due to the numerous iterations required for maker discovery, 2‐4 weeks (depending on the nature of dataset) were needed to determine the signature of a single study.

### 2.3 Assessing the consistency of gene signatures identified from independent datasets

The signatures derived from nine independent studies (Table 1) were analyzed by consistency scores (*CSs*) to evaluate the consistency among signatures identified from independent datasets. The *CS* was new metric quantitatively assessing the consistency of signatures discovered from multiple independent studies.^56^, ^57^ A larger value of *CS* indicates that a greater number of DEGs were shared among those independent studies. As reported, Student's *t* test corrected by *Benjamini‐Hochberg* algorithm^14^ and significant analysis of microarray (SAM)^20^, ^58^ have emerged as the most popular approaches employed to discover SZ signatures. Therefore, the *CS* of the new strategy was compared with that of these popular methods.

### 2.4 Analysis of the reproducibility of gene signatures identified from independent datasets

The performances of one study in predicting the SZ outcome of another and vice versa were critical criteria for assessing the reproducibility of the signatures identified from independent datasets.^59^, ^60^, ^61^, ^62^ Thus, each of the nine independent datasets (Table 1) was initially selected and used to identify SZ signature. Then, the identified signature was used to construct the SVM classifier, and the resulting model was optimized using five‐fold cross validation. Next, the reproducibility of the signature identified from each independent study was assessed by predicting the SZ outcomes of the remaining eight studies (Table 1). Two popular metrics (accuracy (*ACC*) and Matthews correlation coefficient (*MCC*)) in biomedical researches^63^ were applied to assess the predictive performances of nine independent studies (the performance of one study in predicting SZ outcomes of another and vice versa). Collective analysis of eight performance values (*ACCs* and *MCCs*) for each independent dataset could systematically reflect the reproducibility of identified signatures. *ACC* indicated the number of successfully predicted true samples divided by all samples in all eight independent test datasets, and *MCC* reflected the stability of the classifier based on the identified signature.^63^ *ACC* and *MCC* ranged from 0 to 1 and −1 to 1, respectively. The higher value of each metric denoted better predictive performance. An *MCC* of −1 represented total disagreement between the predicted results and independent test dataset, 0 denoted no better than random prediction, and 1 indicated a perfect prediction. As *t* test and SAM were popular methods for discovering SZ gene signature, the reproducibility of the strategy proposed in this study was compared with that of these two popular methods in Section 31.

### 2.5 Elaborating the role of the identified SZ Signature based on enrichment analysis

An enrichment analysis of identified signature was conducted to identify the significantly overrepresented gene ontology (GO) terms such as the biological process, the molecular function, the cellular component, and the KEGG pathways based on hypergeometric test (*P*‐value <.05) provided by gene set enrichment analysis (GSEA).^64^ Based on the comprehensive literature review of GO term and KEGG pathway known to play key roles in SZ, the enriched terms and pathways were applied to reveal the mechanism underlying the cognitive dysfunction in patients with SZ. Moreover, the identified SZ signature was expected to contain a substantial percentage of SZ‐related genes.^65^ Here, a comprehensive literature review was thus performed to investigate the relevance of the signature to the etiology of SZ.

## 3 RESULTS AND DISCUSSION

### 3.1 Consistency among the SZ signatures discovered from multiple independent datasets

Based on the novel strategy developed here, gene signatures were identified from nine independent studies (Table S2). As shown, the total numbers of genes in these signatures varied from 111 to 119. Meanwhile, Student's *t* test (corrected by the *Benjamini‐Hochberg* algorithm) and SAM were applied to discover the gene signature. By selecting the top‐ranked genes (top 100 as frequently applied and widely accepted in DEGs study^66^), a variety of signatures were identified by the Student's *t* test (Table S3) and SAM (Table S4). The *CS* values have been frequently used for quantitative evaluation on the consistency among the signatures discovered from multiple independent datasets.^56^, ^67^, ^68^, ^69^ Therefore, based on the signatures identified from nine independent datasets, the *CS* for each method was calculated. As shown in Table 2, the *CSs* among the nine signatures discovered by the new strategy, *t* test, and SAM were 429, 50, and 82, respectively. This result indicated a substantial increase in the consistency of signatures discovered by the new strategy compared with those two popular methods.

**Table 2:** The reproducibility of two popular feature selection methods (Student's *t* test and SAM) and the new strategy proposed in this study

Eight datasets used as the test dataset	Measure	This study	Student's *t* test	SAM
Consistency score among nine signatures discovered by different methods		429	50	82
B: *Schizophr Res*. 77:241‐252, 2005	ACC (%)	77.4	56.7	60.0
	MCC	0.53	0.15	0.21
C: *Schizophr Res*. 161:215‐221, 2015	ACC (%)	64.4	69.5	64.4
	MCC	0.36	0.45	0.36
D: *Brain Res*. 1239:235‐248, 2008	ACC (%)	75.9	63.0	61.1
	MCC	0.52	0.28	0.25
E: *Mol Psychiatry*. 14:1083‐1094, 2009	ACC (%)	66.0	68.1	59.6
	MCC	0.38	0.43	0.23
F: *Proc Natl Acad Sci USA*. 102:15533‐8, 2005	ACC (%)	64.4	51.1	53.3
	MCC	0.35	0.16	0.24
G: *PLoS One*. 10:e0121744, 2015	ACC (%)	87.5	68.8	62.5
	MCC	0.76	0.46	0.38
H: *BMC Psychiatry*. 8:87, 2008	ACC (%)	85.0	65.0	65.0
	MCC	0.72	0.45	0.45
I: *Neuropsychopharmacol H*. 10:9‐14, 2008	ACC (%)	73.3	66.7	66.7
	MCC	0.44	0.39	0.29

Notes

The consistency and reproducibility were assessed using *CS*s among gene signatures discovered from nine independent datasets and the *ACCs* and *MCC*s for study A (with the largest sample size) to the remaining eight datasets (Table 1).

Increase in consistency of signature identification might improve the reliability and accuracy of identified markers.^30^ Therefore, it was of great interest to assess the predictive performances of those three methods on independent test dataset. Herein, one of the nine independent datasets (Table 1) was selected and used to identify the SZ signature and was then used to construct the SZ classifier. By predicting the SZ outcomes of the remaining eight studies, the reproducibility of the signature identified from each study was assessed. Taking the *study A* in Table 1 as an example, its prediction performances for the remaining datasets were illustrated in Table 2. The *ACCs* for this novel strategy, *t* test, and SAM ranged from 64.4% to 87.5%, 51.1% to 69.5%, and 53.3% to 66.7%, respectively, and *MCCs* were from 0.35 to 0.76, 0.15 to 0.46, and 0.21 to 0.45, respectively. A substantial improvement in the performance of new strategy was observed compared with traditional ones. The predictive performance of one study on the SZ outcome of another was reported to be a key criterion for evaluating the reproducibility of the signatures identified by different datasets.^59^, ^60^ The predictive performance (both *ACCs* and *MCCs*) of all nine studies on the remaining eight independent datasets must be assessed to achieve the comprehensive assessment of the reproducibility of the method.

### 3.2 Reproducibility of the gene signatures identified from multiple independent datasets

**Figure 1:**
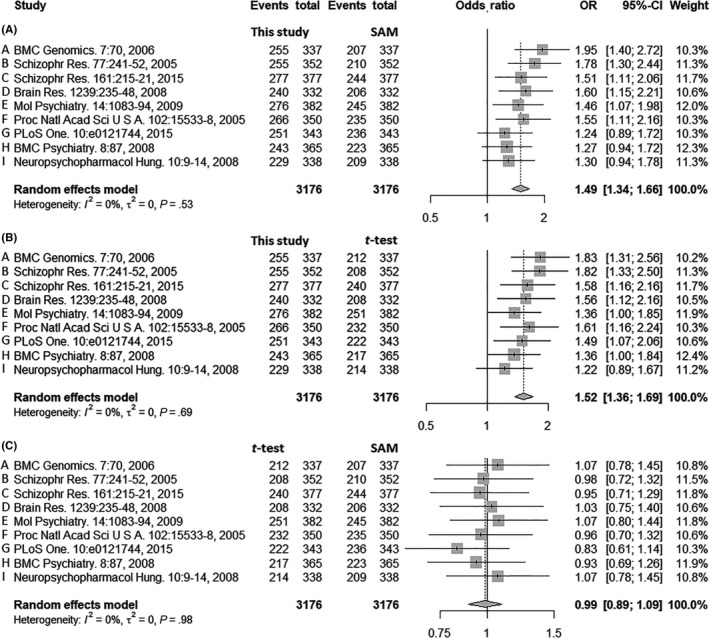
The effect of feature selection methods on reproducibility. Comparisons between A, the newly proposed strategy of this study and SAM; B, this study and Student's *t* test (*t* test); and C, *t* test and SAM are shown. The size of the square indicates the relative weight assigned to the corresponding study in this analysis. The error bars represent 95% confidence interval of the effect. The analysis revealed significant increase in reproducibility when new strategy was employed compared with traditional methods, as shown in (A) and (B), while no significant difference in reproducibility was observed between *t* test and SAM

The predictive performance of nine studies was assessed using *ACCs* and *MCCs* to achieve comprehensive evaluation of methods’ reproducibility (Table 2 and Table S5). Based on the result, forest plots (Figure 1) were drawn to depict the effects of different methods (new strategy, *t* test, and SAM) on reproducibility. Forest plots have been widely adopted in current analyses to discover SZ risk alleles^45^ or assess the drug response rates in patients with SZ.^49^, ^50^ In this study, the comparisons between the new strategy and SAM, between new strategy and *t* test, and between *t* test and SAM were shown in Figure 1A‐C, respectively. The odds ratios (ORs) for those nine independent studies (A‐I) were calculated by random effects models. On one hand, Figure 1A, 1 revealed large and significant overall average effect sizes for the comparison between the new strategy and SAM (OR = 1.49, 95%‐CI [1.34; 1.66]) and between the new strategy and *t* test (OR = 1.52, 95%‐CI [1.36; 1.69]), which indicated the significant increase in the reproducibility by employing the new strategy compared with traditional feature selection method. On the other hand, small and nonsignificant effect size was observed between t test and SAM (OR = 0.99, 95%‐CI [0.89; 1.09]; Figure 1C), which indicated that statistically significant difference in reproducibility was not observed between *t* test and SAM. As shown in Table 1, nine studies were ordered and labeled (A‐I) by their sample sizes. Clear decreasing trend in the ORs was observed as the sample size decreased (from 1.95 to 1.30 in Figure 1A; from 1.83 to 1.22 in Figure 1B). Based on these results, the reproducibility of the new strategy was found to be dependent on the sample size of a specific study.

**Figure 2:**
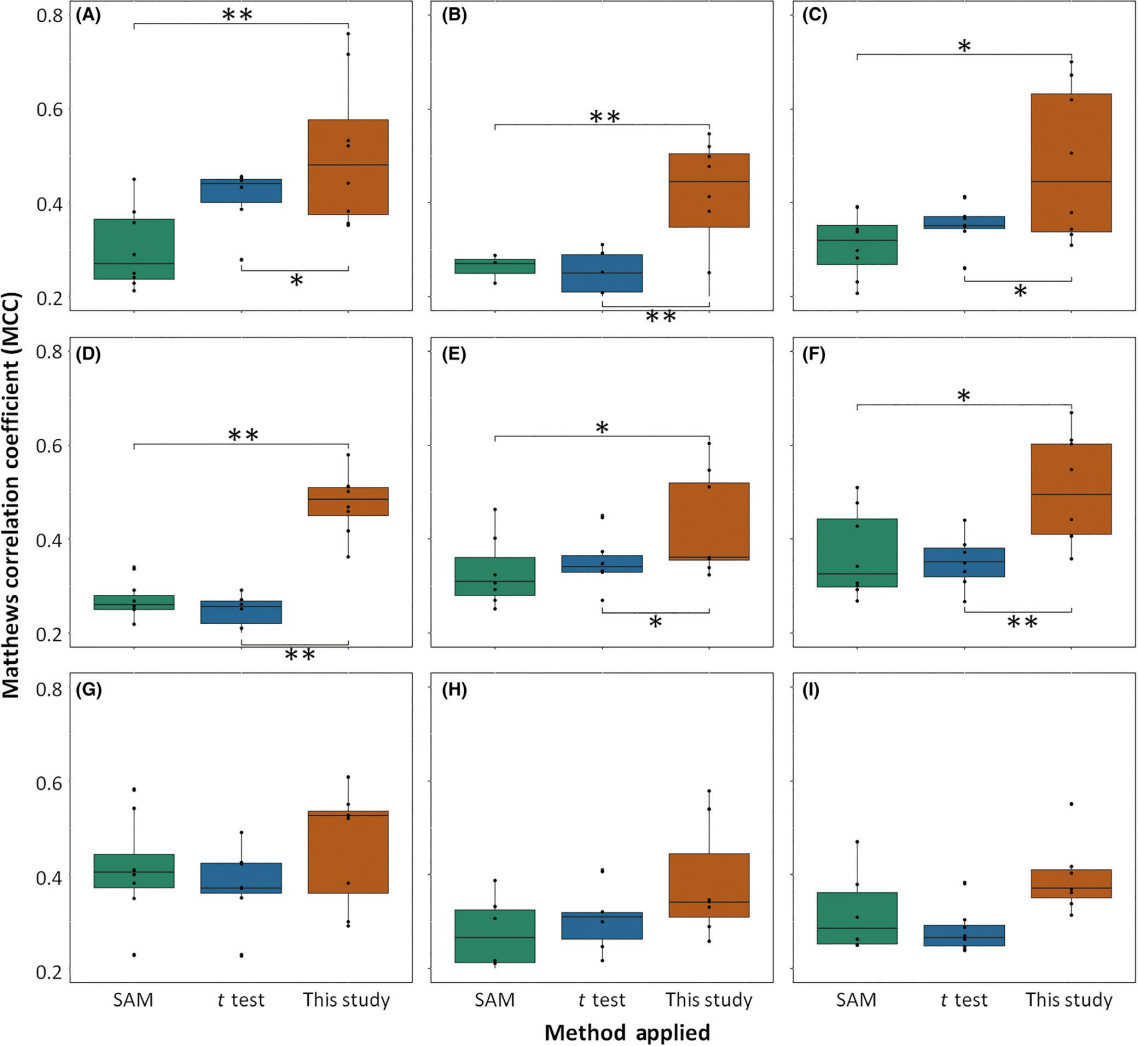
Reproducibility assessed by *MCCs* for each of the nine studies (A‐I) to the remaining eight. The statistical significance of differences among the three methods (this study, *t* test, and SAM) was calculated, and significant differences were observed (* and ** indicated the *P*‐values <.05 and <.01, respectively). The IDs of the nine studies (A‐I) are defined in Table 1

The predictive performance (both *ACCs* and *MCCs*) of all nine studies for the remaining eight independent datasets was assessed for further comparing the reproducibility of the three methods, and the results were presented in Figure S2 and Figure 2; statistical significance of the differences (*P*‐values) among methods was also calculated. As shown in Figure S2, the significant differences (*P*‐value <.05) in *ACCs* for the first six studies (A‐F) were observed between new strategy and traditional ones, but the difference in *ACCs* for all nine studies between *t* test and SAM was not significant. Regarding the *MCCs*, the results were similar to that of the *ACCs*, with significant differences in *MCCs* for the first six studies observed between the new strategy and traditional methods and a lack of statistically significant difference between *t* test and SAM for all nine studies (Figure 2). These results were highly consistent with the data presented in Figure 1, which revealed a significant enhancement in reproducibility when the new strategy was employed. For those studies with relatively small sample sizes (G‐I), although a statistically significant difference (*P*‐value ≥.05) was not observed between the new strategy and traditional methods, the median values (*ACCs* and *MCCs*) obtained by the new strategy were consistently higher than the values obtained using traditional ones in all three studies (G‐I). Moreover, *MCC* was reported as a powerful measure for evaluating the reproducibility due to its complete consideration of the testing data,^63^, ^70^ Figure 2 could therefore be used as another line of evidence confirming the increased reproducibility of the new strategy.

### 3.3 Discovery of the SZ gene signature with enhanced reproducibility

Since the reproducibility of the new strategy depended on the sample size of the studied dataset, six studies with >40 samples (A‐F) were further selected to determine the SZ signature with enhanced reproducibility. The signature of high reproducibility was essential for revealing the molecular mechanism underlying the etiology of SZ.^29^, ^30^ In this study, the gene markers identified by ≥50% of these selected studies (A‐F) were ultimately chosen as the SZ signature of enhanced reproducibility. As a result, 33 DEGs (Table S6) were identified, and the relevance between each DEG and the molecular mechanism underlying the etiology of SZ was comprehensively reviewed based on published studies (Table S7). Twenty‐five of those 33 DEGs were closely related to SZ or its associated cognitive dysfunction. The high percentage (75.8%) of DEGs related to SZ further reflected the reliability of the new strategy.

Additionally, the top 10 ranked GO terms (biological process, molecular function, and cellular component), in which those 33 DEGs were enriched, were listed in Table S8 and the hypergeometric test *P*‐values based on the GSEA^64^ were provided. The response to the stimuli of patient with SZ was attenuated compared with control subjects,^71^ and the positive affects played an important role in regulating the cognitive control.^72^ This result was in accordance with the top‐ranked biological process shown in Table S8 (BP1: positive regulation of response to stimuli). Additionally, increased dopaminergic synaptic transmission and spillage into the extracellular space were reported to be closely associated with SZ,^73^ and an enlargement of extracellular space was frequently observed in the patient of cognitive impairment.^74^, ^75^, ^76^, ^77^ These were consistent with the top‐ranked cellular components shown in Table S8 (CC1: extracellular space). Moreover, the *transition metal ion binding* (MF1 in Table S8) was reported to be significantly associated with SZ.^78^ Furthermore, the enrichment analysis of pathways using those 33 DEGs identified two pathways: (a) the neurotrophin signaling and (b) natural killer cell‐mediated cytotoxicity. The neurotrophin signaling was found substantially related to SZ,^21^, ^22^ and natural killer cell‐mediated cytotoxicity was key for the cognitive deterioration.^24^ Enrichment analysis of transcription factor binding sites based on the 33 DEGs identified two transcription factors as overrepresented, both of which were SZ‐related (Table S9). In summary, this analysis confirmed that the reproducibility of the identified signature was significantly enhanced.

### 3.4 Limitations

The enhanced reproducibility of the newly constructed strategy was primarily derived from its numerous iterations required for marker discovery. The entire calculation process was performed on an HPC server with 768 GB RAM and CPU E7‐8168 × 24 cores and accelerated by a GPU NVIDIA Tesla K80. However, 2‐4 weeks (depending on the nature of studied dataset) were required to determine SZ signature for single study. Thus, this AI‐based strategy was very time‐consuming and relied heavily on the performance of the applied computing server. Thus, further study should be conducted to improve the programming algorithm and server architecture (such as *parallel computing*) for enhancing computational efficiency.

## 4 CONCLUSIONS

The SZ signature identified in this study by new strategy exhibited significantly enhanced reproducibility compared with that of the traditional methods in current SZ studies. Thus, this study not only provided a new strategy for enhancing the reproducibility of SZ study, but also identified several DEGs as candidates for revealing the molecular mechanism underlying the etiology of SZ.

## CONFLICT OF INTEREST

The authors declare no conflict of interest.
