# Supplementary material for: Identification of the gene signature reflecting schizophrenia’s etiology by constructing artificial intelligence‐based method of enhanced reproducibility
Source: CNS Neurosci Ther. 2019 Jul 27;25(9):1054–63. doi: 10.1111/cns.13196 (PMC6698965; doi:10.1111/cns.13196)
Supplement: Supplementary file 1 [file CNS-25-1054-s001.pdf]

**Supplemental Materials for:**

# Identification of the Gene Signature Reflecting Schizophrenia's Etiology by Constructing New Method of Enhanced Reproducibility

Qing-Xia Yang[1,2], Yun-Xia Wang[1], Feng-Cheng Li[1], Song Zhang[1], Yong-Chao Luo[1], Yi Li[1], Jing Tang[1,2], Bo Li[2], Yu-Zong Chen[3], Wei-Wei Xue[2], Feng Zhu[1,2]

[1] College of Pharmaceutical Sciences, Zhejiang University, Hangzhou 310058, China

[2] School of Pharmaceutical Sciences, Chongqing University, Chongqing 401331, China

[3] Bioinformatics and Drug Design Group, Department of Pharmacy, National University of Singapore, Singapore 117543, Singapore

**Correspondence**: Feng Zhu, Lab of Innovative Drug Research and Bioinformatics, College of Pharmaceutical Sciences, Zhejiang University, Hangzhou 310058, China (zhufeng@zju.edu.cn)

**Funding information**

The National Key Research and Development Program of China (2018YFC0910500), National Natural Science Foundation of China (81872798), Innovation Project on Industrial Generic Key Technologies of Chongqing (cstc2015zdcy-ztzx120003), and Fundamental Research Funds for Central Universities (2018QNA7023, 10611CDJXZ238826, 2018CDQYSG0007, CDJZR14468801)

**Short title**: Reproducible SZ Gene Signature

**Supplementary Figure S1.** New strategy constructed for consistently discovering the SCZ gene signature and the combined analysis procedure applied to assess the reproducibility of the feature selection methods. AI: artificial intelligence; ACC: accuracy; MCC: matthews correlation coefficient.

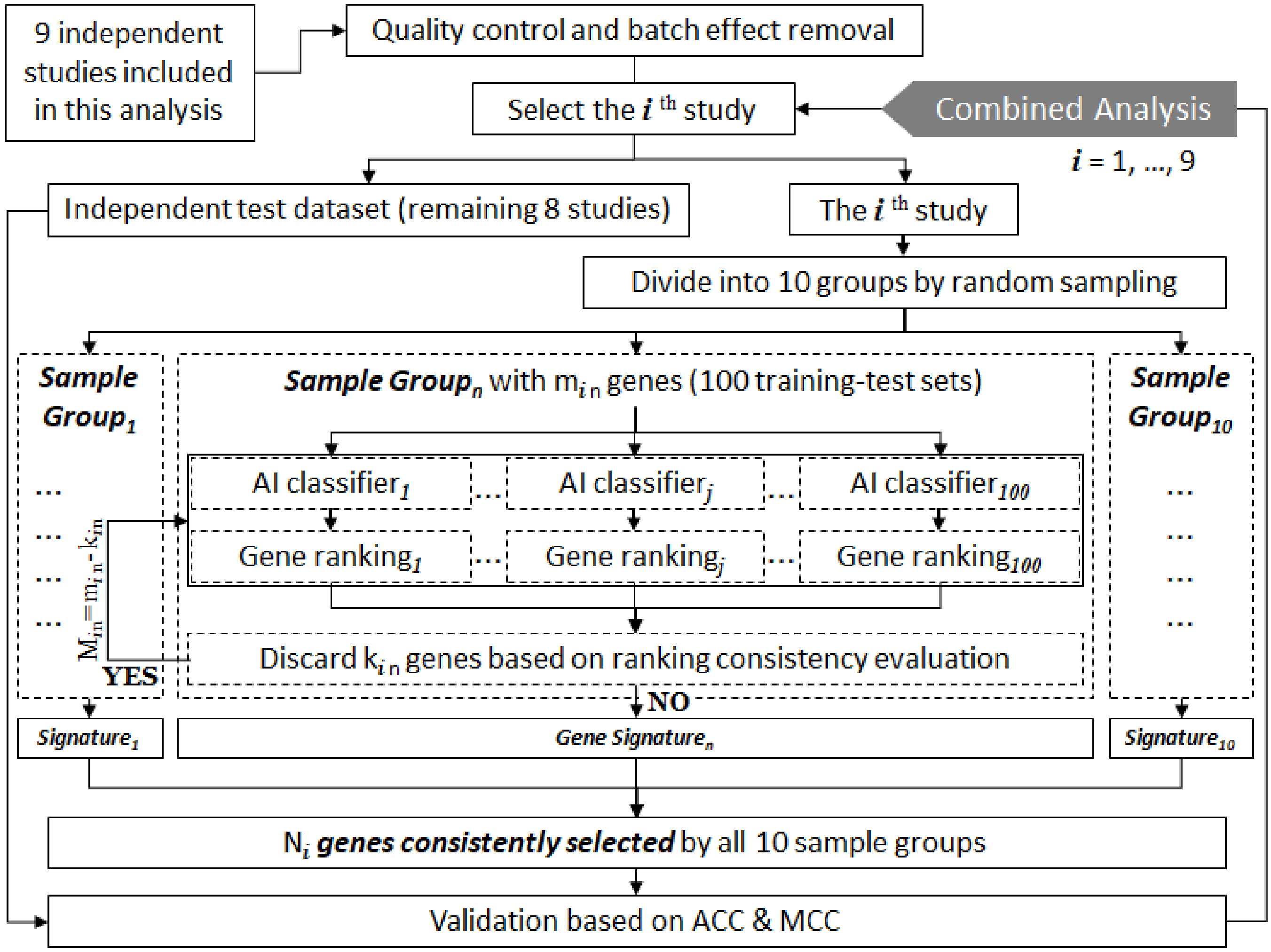

**Supplementary Figure S2.** The reproducibility assessed by the accuracy (*ACC*) of each of the nine studies (A-I) on the remaining eight independent studies other than them own. Statistical differences among three methods (this study, *t*-test and SAM) were calculated, and significant differences were indicated (*p*-values < 0.05 and < 0.01 were denoted by * and **, respectively). The IDs of the nine studies (A-I) were defined in **Table 1**.

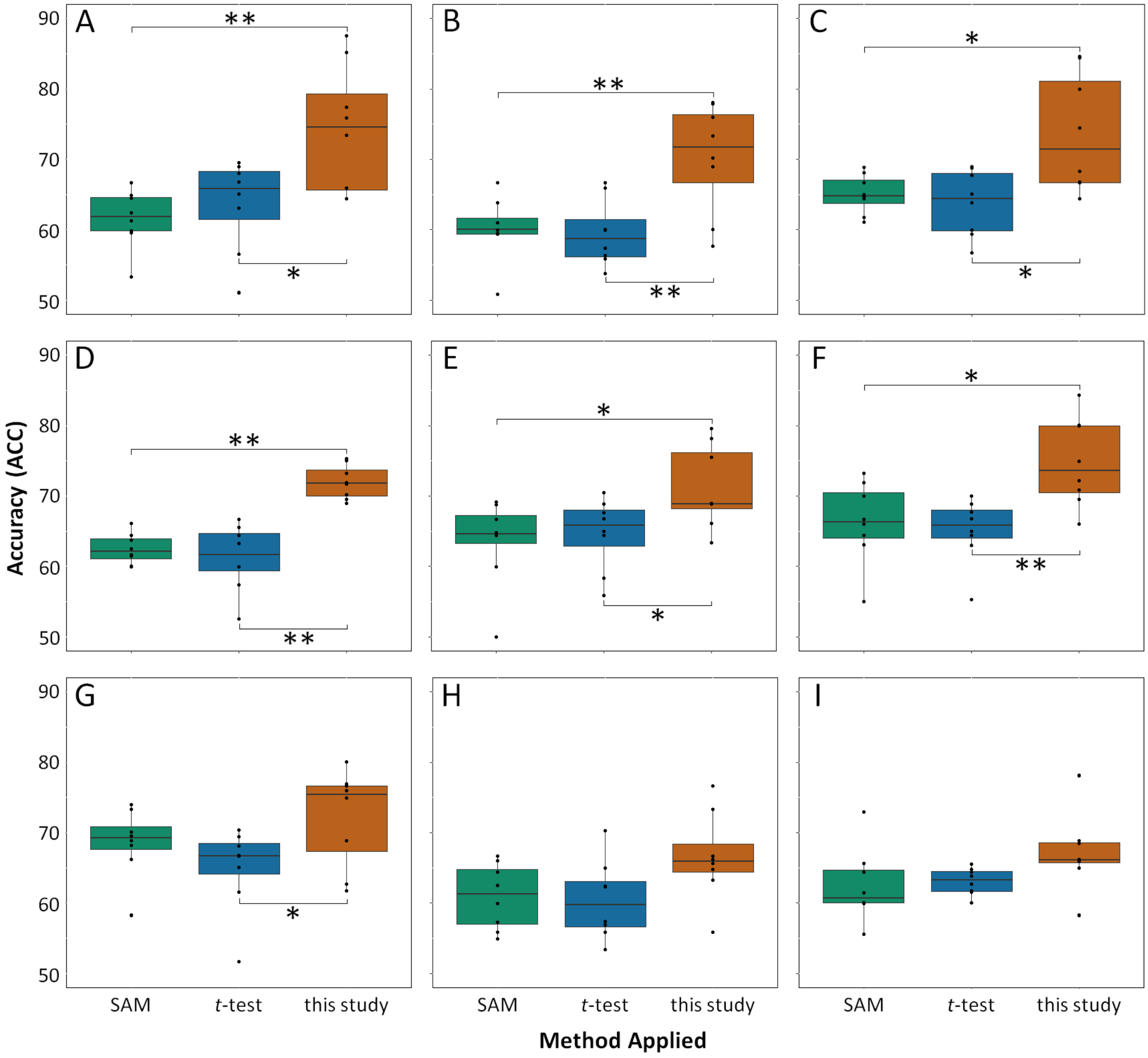

**Supplementary Table S1.** The searching history and the dataset inclusion for each of those seven electronic databases (GEO, SMRI, HBB, PubMed, PsycINFO, Embase and Cochrane). *First*, the total numbers of resulting records by the direct keyword search in the libraries of GEO, SMRI, HBB, PubMed, PsycINFO, Embase and Cochrane equaled to 4,256, 20, 1, 505, 942, 1,346 and 13, respectively. *Second*, the numbers of resulting records by following the five sequential criteria as described in the 2nd paragraph of **Materials and Methods** were provided. *Third*, the numbers of datasets passing five criteria for the libraries of GEO, SMRI, HBB, PubMed, PsycINFO, Embase and Cochrane equaled to 4, 2, 1, 9, 0, 8 and 0, respectively. *Finally*, nine independent microarray studies were collected and included in this analysis by removing the duplicates across all electronic database.

| No. of Records under the Multiple Searching Criteria Applied to Seven Popular Databases | GEO | SMRI | HBB | PubMed | PsycINFO | Embase | Cochrane |
|---|---|---|---|---|---|---|---|
| **Step 1**: Keyword Search[a] | 4,256 | 20 | 1 | 505 | 942 | 1,346 | 13 |
| **Step 2**: Organism *Homo Sapiens* | 4,171 | 20 | 1 | 412 | 529 | 1,140 | 13 |
| **Step 3**: Data Type *Expression Profiling by Array* | 87 | 20 | 1 | 279 | 54 | 210 | 11 |
| **Step 4**: Brain Locus *Prefrontal Cortex* | 10 | 11 | 1 | 77 | 22 | 58 | 1 |
| **Step 5**: Two *Distinct Sample Groups* | 9 | 11 | 1 | 37 | 0 | 47 | 0 |
| **Step 6**: Availability of *Raw Dataset* (CEL file) | 4 | 2 | 1 | 9 | 0 | 8 | 0 |
| **Step 7**: Datasets after Removing the Duplicates | **Nine Independent Microarray Studies (listed in Table 1)** | | | | | | |

[a] the keyword search was conducted using the "schizophrenia AND (gene expression OR microarray OR transcriptomics)"

**Supplementary Table S2.** SCZ gene signatures identified from nine independent studies using the newly proposed strategy.

| **ID: Dataset Studied (No. of DEGs Identified)** | **The DEGs Identified from Each of the Nine Independent Studies** |
|---|---|
| A: *BMC Genomics*. 7:70, 2006 (112) | ADIRF; ADM; ALAD; ALB; AMFR; ARHGEF9; ARMCX5; ARRB2; ASH2L; ATP8A2; CCBL2; CDV3; CNGB3; COL4A2; COX7A1; COX7A2; CRB1; CRHBP; DAG1; DGCR9; DHX16; DTX4; ECHDC2; EIF6; ELP4; EXOSC2; FBLN5; FJX1; GAS7; GCC1; GEMIN6; GGTLC1; GOT2; GPR35; GSC2; GSS; HLA-DPA1; HMGN1; HSD11B1; HSPB3; IQCA1; ITGAV; JMJD6; KCNA3; KCNQ4; KCNS1; KDM5D; KIAA0556; LBH; LOC100129973; LPP; LSM4; MAD2L1BP; MCCC1; MFSD1; MICAL2; NDUFA9; NFE2L2; NHP2; NKIRAS2; NOP14-AS1; NPTN; NQO1; NSUN5P1; NXF1; PAIP2B; PCSK6; PDE1A; PEX14; PLCG1; PLD2; PSMA1; PSMB6; RAB33A; RAB3A; RAMP1; RANBP10; RAPGEF1; RASSF2; RBFADN; RGN; RGS7; RMND5B; ROBO3; ROR1; RPL36; RPP25; RRP8; SCN1B; SCN8A; SELENBP1; SLA; SLC39A1; SMARCD2; SMPX; SOX30; SUPT4H1; TAC1; TBC1D2B; TNFSF10; TRPM3; TSPAN13; TSPYL5; TTC19; TTYH1; TXNL4B; UTY; UXS1; VASH2; WFS1; WSB2; ZNF593 |
| B: *Schizophr Res*. 77:241-52, 2005 (115) | ANKS1A; ARHGEF6; ARMCX5; ARPP19; ARRB2; ASH2L; ATG101; ATP1A3; ATP6V0D1; BABAM1; BAG3; C14orf159; CARS2; CCBL2; CCNB1IP1; CCT8L2; CD79B; CDK18; CIB1; CMTR1; CNN3; CNTRL; CPNE7; CRB1; CSN3; CTGF; CUTC; DECR1; DGCR9; EDEM1; EIF3B; ENDOG; EPB41L3; ESYT1; F8; FAM107A; FBXO46; FLCN; FOXO1; GAS7; GLIPR1; GLRX; GNE; GNG12; HSD11B1; HSPB1; IFT20; IGFBP2; IL4R; ITGAV; KCNS1; KDM5D; LDOC1; LIPG; LYRM4; MED15; MEFV; MGP; MRPS7; MT1M; MUC2; MYL5; NCAN; NDRG1; NEFH; NEUROD6; NFU1; NHP2; NOTCH1; NT5C2; NTRK2; NXF1; ORMDL2; PCP4; PEX14; PKN1; PLEKHF1; POLA2; PPA1; PPCS; PPP1R26; PPRC1; PSMD7; PSRC1; PSTPIP2; RAB33A; RAMP1; RBMS1; RGN; RPL36; RYR3; S100A12; S1PR1; SCN1A; SDC4; SEPT2; SEPT4; SERPINE2; SLC25A12; SLCO1C1; SOX9; SST; SUSD6; TAC1; TCEA1; TGFBR2; TMEM2; TNFSF13; TOLLIP; TUBB6; UBE2E3; VAMP1; WARS; ZHX2; ZNF134 |
| C: *Schizophr Res*. 161:215-21, 2015 (118) | ADM; AHCYL1; AK5; ALDH1L1; AMZ2; ANKRD1; ANKS1A; APOO; ARPC1A; ATP1A2; ATP5G1; ATP6V0A1; CADM1; CEP70; CHPT1; CMC2; CNTRL; CP; CX3CR1; DAG1; DHRS3; DUSP14; DUSP6; EBP; ELOVL2; ERF; EZH1; FABP3; FHOD3; FTO; GGTLC1; GLUD1; GMPR2; GNAL; GPI; GPX4; GSTA1; HIF3A; HOXB9; HSD11B1; IMP4; KDM5D; KDM7A; KIAA0556; LAPTM4B; LCN2; LRP2BP; LRRC31; LYRM4; MAEA; MCCC1; MCL1; MEF2C; MFSD1; MT3; MTCH2; MUC2; MYBPC1; NCAN; NDUFA8; NDUFA9; NINL; NMU; NOP14-AS1; NOS3; NOTCH2NL; NPTX2; NXF1; OFD1; OTUD4; PAQR6; PARP2; PBX2; PCYOX1L; PDZD2; PEX11B; PKD2L1; PLCG1; PLIN3; PMS2P3; POLR1B; PPP1R2; PRR5; PSMA2; PSMB3; PTPN3; RBMS1; RBMX2; RERGL; RNF126; ROBO3; RPS6KA2; RREB1; RUFY1; S100A12; S100A8; SCG5; SCN1B; SLC25A17; SNRPB; SNX5; TCP11; TIMM10; TNFSF10; TOPBP1; TRAT1; TXNL4A; TXNL4B; VAMP1; VGF; VILL; VPS72; WFS1; ZCCHC6; ZFAND3; ZNF134; ZNF337; ZNF593 |
| D: *Brain Res*. 1239:235-48, 2008 (111) | ACKR3; ACLY; AFF1; ALB; AMZ2; ANKRD1; APOBEC1; ATRNL1; BCAN; C1QTNF3; C2CD2; CCBL2; CCDC86; CDC42; CDV3; CHST15; COL4A2; CYP26B1; CYR61; DDIT4; DDX25; DHX16; DIDO1; DOCK9; DYNLT3; ECSIT; EFR3A; EIF2AK1; ENDOG; ERF; ETS2; FAM13B; FAP; FARP2; FOCAD; FOXG1; GALNT10; GCC1; GDF10; GGCT; GNAL; GNPDA1; GPR63; GSC2; HIST1H2BO; HMGB2; HSD17B11; IGFBP2; INHBB; IQCA1; ISG15; ISLR; ITM2A; KCNB1; KIAA0930; KLF7; KRTAP4-7; LIPG; LPL; LRRN3; MED12; MEFV; MFAP3L; |

| | |
|---|---|
| | MFSD1; MGP; MICAL2; MRPL22; NEMP1; NME5; NPY; NT5C2; NUP214; OAT; OPN3; OSBPL10; OTUD4; PCSK1; PDZD2; PLEKHM1; PLXNA3; PNMA3; PSMB6; PSMD14; PTK6; PXDC1; RAPGEF3; RBPJL; REEP1; RFPL1S; RGN; ROR1; RPL36; SCN8A; SERP1; SHROOM2; SLC1A1; SNAP91; SNF8; SOX30; SPRY1; TAC1; TBC1D2B; TFIP11; TGFB3; TIMM10; TNFSF10; TOMM20; TRAT1; TTLL1; TYRP1; ZNF750 |
| E: *Mol Psychiatry*. 14:1083-94, 2009 (119) | ACTR1A; ACTR1B; ADAM19; AKTIP; AMELY; AMFR; ANKMY2; ANKRD1; ANP32B; APOE; ARHGAP19; ARHGEF40; ARL4C; ATP6V1A; BAG3; BCAN; BDH2; C19orf60; CACNA1D; CAP2; CCBL2; CMTR1; CNNM4; CP; CRYBG3; CTGF; CYP26B1; DAPP1; DGCR9; DHODH; DOC2A; DOCK9; DPYSL4; DTX4; DUSP9; EDEM1; EIF2AK1; ENGASE; EXOG; FARSA; FBXL7; FER1L4; FJX1; GRIN2C; GSC2; GSS; HIST1H2BO; HOXB9; HSPB1; IL1A; JCHAIN; JMJD6; KCNA3; KCNQ4; KDM5D; LCN2; LIMS2; LRRC31; MAP4K4; MEF2D; MGP; MICAL2; MLNR; MRPS18B; MRPS22; NEFH; NFYA; NNMT; OAT; OSBPL10; P4HA1; PALLD; PAQR6; PCP4; PCSK6; PCYOX1L; PDAP1; PDE6H; PFKP; PIK3R4; PLIN3; PLSCR4; PLXND1; PMAIP1; PMS2P4; PNMA1; PRKD2; PSMB3; PSMD14; PSRC1; RANBP10; RAPGEF1; RERGL; RPS6KA2; RPS6KA5; S100A8; SHTN1; SLC25A4; SMARCD2; SOX30; SPCS1; STK17B; STMN2; TCP11; TIAL1; TJAP1; TMPRSS3; TRAT1; TSN; TTYH1; TULP3; TXNL4B; TYRP1; UCP1; UTY; VASH2; YPEL5; ZBTB40; ZNF750 |
| F: *Proc Natl Acad Sci*. 102:15533-8, 2005 (111) | ABCA1; ABCG2; ACO1; ADGRA3; ADM; ALAD; ALB; APBA3; ARHGAP5; ARHGEF10; ARMCX5; ASAH1; ATP5G1; BCAS1; C1QTNF3; C2CD2; CACNA1D; CAMLG; CBFA2T2; CCDC53; CCNB1IP1; CMC2; CNN3; CNTRL; CP; CPQ; CRB1; CRHBP; CX3CR1; CYR61; DNAAF5; DUSP14; EFEMP1; ENAH; ENPP1; EPHX1; EXD2; EXOG; EXOSC2; FAP; FARP1; FBXO42; FBXO46; FER1L4; FGFR3; FJX1; GABARAPL1; GABRB1; GDAP1; GDF10; GPR63; HMGN1; HOXB9; HR; HSPBP1; HTRA2; IL22RA1; KCNA3; KCNJ4; KCNS3; KLHDC3; LPL; LRRC20; MAD2L1BP; MAP2; MARCH3; MCCC2; MEF2D; MEFV; MLYCD; MRPL46; MTCH2; NDUFA8; NINL; NMU; NOL3; OXTR; PCBP4; PCSK6; PDAP1; PDE1A; PDE6H; PEX14; PKP4; PLCG1; PLEKHM1; POMZP3; PPA1; PRPF19; PWP1; RAPGEF1; ROCK1; RRP8; RYR3; S100A8; SCN1B; SHROOM2; SST; SUSD6; TESPA1; TMEM127; TMEM2; TNFSF10; TRAK1; TRIL; TXNL4A; UNC45A; UXS1; VEGFA; VILL; WDR6 |
| G: *PLoS One*. 10:e0121744, 2015 (111) | ADM; ANKLE2; ANKS1A; APOBEC1; ARHGEF40; ATP6V0A1; BABAM1; BACE2; BAG3; BCL6; C6orf106; CAP2; CDKL3; CDV3; COPS3; CTRC; DDIT4; DOLPP1; EDN3; ELOVL5; ENDOG; ERCC1; ETS2; EXTL2; FABP3; FAM13B; FARP1; FGF1; FLJ11710; FOXG1; GCC1; GNB1; GNB5; GNG12; GOT2; GPR63; HMGB2; HMGCR; HMGN1; HMOX1; HMP19; IFIT1; IGFBP6; IL1A; INPP4A; INPPL1; IQCA1; ISLR; KCTD15; KLF7; KLHDC3; KRTAP4-7; LAMTOR5; LEPROT; LRRN3; LYZ; MARCH3; MCCC2; MX1; MXRA8; MYL5; MYO5A; NDRG4; NEFH; NELL2; NINL; NOL3; NOP10; NSG1; NUMA1; PCDHGA1; PCYOX1L; PJA1; PKP4; PLXDC1; POMZP3; POP1; PRSS2; PVALB; RBMS1; RBMX2; RERGL; RND1; RRP8; S100B; SCN1B; SERPINE2; SH3GL2; SNF8; SOX30; STXBP1; TBC1D2B; TCP11; TESPA1; TGFBR2; TIAL1; TMOD1; TNFSF10; TOLLIP; TRAF4; UBAP1; UCP1; UNC45A; UQCRC1; VASH2; WDR6; WWC3; ZBTB40; ZMAT4; ZNF24; ZXDC |
| H: *BMC Psychiatry*. 8:87, 2008 (111) | ABCG4; ACAT2; ACIN1; ALDH7A1; AMFR; ANP32B; APOLD1; ARHGAP19; ARRB2; ATP1A3; ATP5G1; BDH2; CAMKK2; CBFA2T2; CCDC25; CCDC86; CCP110; CEBPD; CFAP20; CNGB3; CPNE7; CRIP2; CX3CR1; DAPP1; DNAAF5; DUSP7; ERBB2IP; EXD2; EXOG; F8; FJX1; FLCN; FTO; GNPDA1; GPD1L; GPX3; GTF2B; HIST1H2BO; HMGCR; IFIT1; IL4R; IQCA1; IQGAP1; KANK1; KCNS3; KIAA0556; KIAA0930; LPAR1; MARCH3; MED15; MICAL2; MLC1; NDFIP1; NDUFA8; NPY; NUMA1; OAT; PAFAH2; PDE1A; PHC1; PHGDH; |

| | |
|---|---|
| | PIH1D1; PJA1; PMP22; POP1; PPA1; PPP3CB; PRKD2; PRR5; PSMD14; PTCH1; PTK6; PTPRN; PTPRN2; PTS; RAB3A; RANBP10; RANBP17; RBFADN; RCAN2; RERGL; RGN; RGS7; RNF19B; RREB1; S100A8; SLC17A7; SLC25A20; SLCO4A1; SMTN; SPON1; SST; SYP; TIAM1; TMEM59L; TMOD1; TNFRSF25; TNFSF10; TNS2; TRAK1; TRAT1; TRIL; TSN; TTC1; TTLL1; TXNIP; TXNL4A; UBAP1; UNC45A; VASH2; ZNF593 |
| I: *Neuropsychopharmacol H.* 10:9-14, 2008 (111) | ABCG2; ACO1; ACTA1; AKTIP; ANKLE2; ANKMY2; ARL4C; ARMCX5; ARPP19; ARRB2; BACE2; BAG3; BCAT2; BCL2A1; BCL6; BTN2A2; CAMLG; CCDC25; CCDC53; CCNB1IP1; CCT8L2; CHP1; CHPT1; CHST10; CNGB3; CRB1; CSE1L; CUTC; CX3CR1; CYR61; DAPP1; DCHS1; DDIT4; DECR1; DHX16; DUSP7; EPB41L3; FARP2; GABRB1; GDPD3; GGTLC1; GLRX; GNAL; GNE; GPC4; GPD1L; GPKOW; GPR35; GSS; GSTA1; HIST1H2BO; HLA-DPA1; IMP4; ISG15; ITM2A; ITPKC; LBH; LMAN2L; LPL; MAPKAPK3; MARCH3; MCCC2; MLNR; MRPS22; MX1; NEUROD6; NIF3L1; NPY; P2RX4; P4HA1; PAFAH2; PARD3; PCMT1; PCSK1; PFKP; PKIG; PLEKHF1; PLEKHM1; PPIH; PSRC1; PVALB; RANBP17; RBM4B; RBMX2; RGR; RMND5B; RNF19B; ROR1; RPP25; S100A8; SDC4; SLC39A1; SLC4A1AP; SMPX; SOX9; ST5; STAT4; SUSD6; TIAL1; TIMM10; TMEM97; TNFSF10; TRAF4; TTC19; TTC28; TXNL4B; UNG; VASH2; VILL; ZCCHC6; ZSCAN9 |

**Supplementary Table S3.** SCZ gene signatures identified from nine independent studies using the Student's *t*-test.

| **ID: Dataset Studied (No. of DEGs Identified)** | **The DEGs Identified from Each of the Nine Independent Studies** |
|---|---|
| A: *BMC Genomics*. 7:70, 2006 (100) | ACOX1; ALDH1L1; ALDH7A1; ATP1B2; ATP5A1; ATP8A2; BASP1; BEX1; BID; CADM1; CCKBR; CCND2; CEP104; COX7A2; CPT2; CRH; CRHBP; DIRAS2; ECHDC2; FABP3; FAM107A; FBXO9; FERMT2; FOXO1; GAD1; GNG12; GOT2; HTRA1; ITPKB; JUP; KIAA0556; KIF3C; KIFAP3; LBH; LDHA; LDHB; MAFB; MDH1; MLLT11; NBEA; NDRG1; NDUFA9; NEFH; NEFL; NEFM; NEK9; NELL1; NEUROD6; NFATC3; NQO1; NSF; NSG1; PANK2; PARP16; PART1; PBXIP1; PCSK1; PGAM1; PHGDH; PIP4K2A; PLCL2; PLIN3; PLXNB1; PPAP2B; PRKCB; PTTG1IP; PVALB; RABIF; RASL12; RCAN2; RGS7; ROBO3; SCG2; SDC4; SELENBP1; SLC9A3R1; SMARCC1; SMYD2; SOX9; SPA17; SST; STMN1; STS; TAC1; TBC1D9B; TBL1X; TIMM17A; TJAP1; TNS1; TP53; TPP1; TSPAN13; TST; TUBB2A; TULP3; TXNL4B; UST; WIPI1; WSB2; ZHX2 |
| B: *Schizophr Res*. 77:241-52, 2005 (100) | ADCY3; AKAP10; ANKRD10; ARRB2; ASCC3; ASH2L; ATG4B; ATM; BCL2L13; BNIP2; BRD1; BRD9; C2orf68; C4orf29; CAB39L; CARS2; CCDC109B; CCNB1IP1; CDK5RAP1; CDKN2C; CIC; CKLF; CMTR1; CNPPD1; CUTC; CXorf57; CYLD; DDX19A; DFNB31; DHX57; DNAJA3; DNAJC1; DSE; DYRK4; EDEM1; ERCC5; FAM49B; FAM86C1; FCGR2A; GAA; GBF1; GEMIN6; GOLGA4; GPN3; GTF2IRD1; HGSNAT; IFT81; IL1R1; KAT5; KDM4C; LAMA5; LGALS9; LSM2; LSR; MDC1; MED15; MRPS18B; NFATC3; NIPA2; NUP37; PEX14; PHC2; PLIN2; PLK3; PPRC1; QRSL1; RAB33B; RALY; RBMS1; RCL1; REV3L; RFC2; RING1; RRNAD1; SCARA3; SCIN; SGSH; SLC25A17; SLC35D2; SLC44A1; SNRNP40; SNX11; SORD; SPIDR; STK3; TFG; TGIF1; THOC1; TIAL1; TMBIM4; TMEM120B; TOR1AIP2; TRAF3IP2; TRPC4AP; TTTY15; UBE2J1; WDR60; ZBTB14; ZMYM2; ZNF175 |
| C: *Schizophr Res*. 161:215-21, 2015 (100) | A2M; AHCYL1; AKAP1; ALDH1L1; ANKRD53; AOC2; ARC; BDKRB1; C17orf80; CALCA; CCND2; CD3G; CDC5L; CETP; CP; CRTAM; CSN2; CYTIP; DAZL; DOC2B; DPYSL3; DUSP1; DUSP5; DUSP6; EDN2; EGR1; EGR2; EGR4; ETV5; F7; FBXW11; FGL2; FKBP5; FMO5; FOS; GDF10; GPR20; GTPBP4; GZMK; HIF3A; HRAS; HSD11B2; HSD3B1; HTRA1; IL25; IL5; KIF13A; KIR3DX1; KRT5; KRT6A; MCM6; MDC1; MMP1; MNS1; MROH9; MT3; MYBPC1; MYOZ1; NOP14-AS1; NOTCH2NL; NR4A3; OFD1; PAPSS1; PAX4; PGLYRP1; PI15; PLIN1; PLVAP; PRKAB2; PROCR; RERGL; RNF34; RPS17; RPS8; SAMHD1; SCGB1D1; SCGB1D2; SCGN; SERPIND1; SLC14A1; SOD3; ST8SIA2; TBX5; TEX2; TMEM231; TMPRSS5; TNFSF10; TRIB2; TRIM15; TRIM24; TRPC3; TTC13; UTRN; VEGFC; WNT7A; ZBTB32; ZBTB6; ZFP2; ZNF250; ZNF8 |
| D: *Brain Res*. 1239:235-48, 2008 (100) | AARS; ACAT2; ACOX1; ADAM23; AKR1C3; APBB1; APOC1; ARHGEF11; ARHGEF2; ARL2BP; ATP2A2; ATP5A1; BEX1; C16orf45; C1R; CAMK2B; CCBL2; CCK; CCNI; CDC42; COL18A1; CRHBP; DCLRE1B; DDIT4; DIO1; DNAJC3; DOPEY2; DRICH1; DYNLT3; ELAVL4; ENO2; EXTL2; FARP2; FBXO9; FZD10; GABARAPL1; GAD2; GOLIM4; GOT1; GOT2; HIST1H1C; HSPA12A; HSPB1; HTR1F; IL10; IL2; INHBB; KIAA0101; LAMB2; LAMB3; LEPROT; LOC441204; LPA; LRPPRC; LYZL6; MAGED1; MBTPS2; MDH1; MPO; MRPS22; NDRG3; NEUROD6; NGFRAP1; NPY; NREP; NRN1; NSF; OPN3; PALLD; PAM; PARD3; PCP4; PFN2; PINK1; PPME1; PRSS12; PVALB; REEP1; RGS7; SLC25A12; SLC7A8; SNAP25; SNAP91; ST3GAL6; STXBP1; SULT4A1; SV2B; SYN3; SYNDIG1; TGIF1; TRIM37; TUBA1B; UBXN2B; VIPR1; VSNL1; YAF2; ZC3H13; ZNF550; ZNF576; |

| | |
|---|---|
| | ZNF839 |
| E: *Mol Psychiatry*. 14:1083-94, 2009 (100) | ABCB9; ACTR1B; ADAMTS8; ALOX5AP; APBA2; ARF5; ARID5B; ATP11B; ATPIF1; B4GALNT1; BAG3; BRINP2; C11orf80; C1QB; CBX4; CCDC82; CCND2; CEP131; CNPY2; COL3A1; COLEC10; COMMD4; CRYBG3; CRYM; CTSW; CYP26B1; DKK1; EED; EIF3M; FMNL1; FMOD; FRZB; GHR; GSS; GSTA3; HEBP2; HPCA; HRH3; HSPB1; IKZF5; INSL3; IRF8; JMJD6; KAZALD1; KCTD12; KDM3A; KIAA0408; KIAA1644; MAPK11; MCM4; MLNR; MOB1A; MPPED2; MPV17; NDUFA6; NEUROD6; NRSN2; OGG1; OGN; P4HA1; PAPOLA; PEX7; PITRM1; PMAIP1; PTK7; PXDN; PYGL; R3HDM4; RABL3; RAPGEF1; RASGRP1; RERGL; RNASET2; S100A8; S100A9; SAMSN1; SARS2; SLC25A24; SLC2A5; SLC30A3; SLCO4A1; SMAD7; SNX6; SPCS1; SSR1; SYK; TIMP4; TMEM184C; TOM1; TRAT1; TRIB2; TRPC3; TSR3; UBE2J1; UGGT2; ZBTB40; ZNF26; ZNF395; ZNF747; ZNRF4 |
| F: *Proc Natl Acad Sci*. 102:15533-8, 2005 (100) | AAGAB; ACO1; ACOX1; ACTC1; ACVR2B; ANKRD11; ANKRD12; APC; ARHGAP25; ARHGEF2; C1GALT1; C21orf59; CADM1; CCS; CEP290; CEP97; CHRDL1; COL16A1; CORO7; CYP20A1; DCAF11; DDAH1; DHX57; DNAAF5; DSN1; DUSP14; DYNC1LI2; ENAH; ERBB4; FABP6; FAM204A; FAP; FARP1; FNBP1L; FNDC3B; FRY; FXR2; GABRA2; GABRB1; GALNT12; GANAB; GNAS; GSK3A; HIRA; HMGA1; HSP90B1; IFT140; IGF1R; KBTBD4; LPL; LRRC37A2; LSG1; MAPKAPK5-AS1; MARC2; MGAT4C; MIPEP; MMP16; MRPL20; MRPS16; N4BP3; NEK1; NOL3; NTM; NTSR2; OPHN1; PCK2; PCSK5; PHF21A; PLCXD1; PLOD2; PPIG; PRKD1; PRPF19; PRRC2C; RABGAP1; RBL2; RETSAT; RMND5A; RPL27A; RUSC2; RXRB; SCAPER; SEC61B; SFXN3; SMIM14; SRI; SRRD; SSBP3; SSPN; STK16; SUPT5H; TESPA1; TMEM47; TMEM50A; TNIK; TPBG; UXS1; WARS2; ZBBX; ZNF419 |
| G: *PLoS One*. 10:e0121744, 2015 (100) | ADAM11; ADRBK2; ARHGEF2; ARHGEF7; ASPH; ATP1B2; ATP1B3; ATP2B2; ATP6V0A1; BABAM1; BASP1; BDH2; CAP2; CASP7; CD81; CHRDL1; COL9A1; DCAF6; DECR1; DENND4C; DNAJC28; DPP4; EDN3; EIF3G; ELOVL5; EPN3; ERLIN2; ETS2; FABP3; FAM65B; FUBP3; GFRA2; GLS; GNA13; GNB5; GNG12; GOT2; GRB2; GRK4; HADHB; HMGN1; HS3ST1; HS3ST3B1; HSD17B7; HSDL2; IFITM3; IGFBP6; IL17RB; ISLR; ITGAV; KLF15; KLF5; LONRF3; MAGOHB; MARK2; MEIS2; METTL7A; MREG; MRPS18A; MTUS2; NOTCH2; NOTCH2NL; PAAF1; PIGA; PIK3CB; PLXDC1; PON2; PRKCI; PTPN13; PTPRZ1; PVALB; RGS6; RRN3; RUSC1; SCN1B; SDC4; SHMT1; SLC19A2; SLC6A16; SLC7A11; SLCO1C1; SMAD1; SNX15; SOX2; SPON1; STK24; SUCO; SYNDIG1; TIAL1; TMPO; TOLLIP; TRAK2; UBR5; UNG; USP3; XPO1; ZC3H13; ZMAT4; ZNF160; ZNF267 |
| H: *BMC Psychiatry*. 8:87, 2008 (100) | ACTN1; ALG5; ATG3; ATP5G1; AVL9; BET1; BORCS6; CBFA2T3; CCDC25; CILP; CKAP5; DCAF16; DENR; DGCR2; DGUOK; DIABLO; DROSHA; EDN1; EIF4G1; ELOVL6; ENOPH1; ERCC5; FAM8A1; FLJ10038; GGT5; GNPDA1; GPR137; GRHPR; GTF2B; HARS2; HDAC9; HEY1; HIBCH; HSF1; IFT52; IKZF3; IL36RN; INSIG1; JAM3; KIAA0232; KIAA0368; KIAA0895; KIAA0930; KIF26B; LACTB2; LYRM4; MAD2L1; MAGEF1; MAPK6; MED4; MED7; MED8; MRPL48; MXD3; MYH9; NKX3-1; NOC3L; OSBPL2; PARN; PDCD10; PIN4; PLA2G16; PPP1CC; PPP3CB; PPT1; PSMB4; PSMD4; RAB5C; RBM15; RBX1; RCAN2; RER1; RHBDF1; RPA1; RPAP3; RRP15; SCUBE3; SEPHS1; SETD2; SGMS1; SMTN; SNRPB2; SNX19; STK39; SUSD5; TBP; TCFL5; THOC7; TMEM176A; TPMT; TTC1; TXN; UBE2O; UROD; VPS26A; YTHDC1; ZBTB24; ZCCHC10; ZYX; ZZZ3 |

| | |
|---|---|
| I: *Neuropsychopharmacol H.* 10:9-14, 2008 (100) | ADAM2; ADAM28; ADH1C; AGL; ALX3; AP4M1; APOA4; ARHGEF2; ARMC6; BCL6; CCL25; CNGB1; COPS5; CSF2; CST1; CTH; CXCR3; CXorf36; CYBB; CYTL1; DCP2; DDX28; DNAH9; EPX; FAM35A; FAM64A; GJB5; GMNN; GNE; GOLGA2P5; GPR182; GRPR; GSTO1; HARS2; HDLBP; HIST3H2A; HLA-DQA1; HOXD9; HSF2BP; ITSN1; KIFC1; KLK11; KRTAP1-3; LARP1; LARP4; LPL; MAFF; MIOS; MNS1; MORN1; MTMR12; MTSS1L; NCF2; NPR1; NR2C2; NSUN3; NUDT7; OGG1; OXSR1; P2RX4; PIKFYVE; PLEK; PLEKHF2; PLOD2; PP13; PROC; PTGER1; RAB8A; RANBP1; RAP2C; RBM4B; RRP1; RSPH6A; SDK2; SELPLG; SERHL2; SESN1; SIX1; SLC39A8; SLCO2B1; SOCS7; SPAG8; STX18; SYNPO; TBXAS1; TCL1B; TM6SF2; TOMM40; TOP2B; TRAM2; TRPM1; TTC17; TUBGCP4; TVP23B; VCX2; WDR62; XAB2; ZIC3; ZNF606; ZNF75D |

**Supplementary Table S4.** SCZ gene signatures identified from nine independent studies using the SAM.

| ID: Dataset Studied (No. of DEGs Identified) | The DEGs Identified from Each of the Nine Independent Studies |
|---|---|
| A: *BMC Genomics*. 7:70, 2006 (100) | ALDH1L1; ALDH7A1; AQP1; ATP1B2; ATP5A1; ATP8A2; BASP1; BEX1; BID; CADM1; CAPZA2; CCKBR; COX7A2; CPT2; CRH; CRHBP; CRYM; DIRAS2; ECHDC2; EMX2; EPHX1; FABP3; FAM107A; FBXO9; FERMT2; FHL2; FOXO1; GAD1; GNG12; GOT1; GOT2; HTRA1; IL17RB; ITPKB; JUP; KIAA0556; KIF3C; KIFAP3; LBH; LDB2; LDHA; LGALS3; MAFB; MDH1; MLLT11; NBEA; NDRG1; NEFH; NEFL; NEFM; NELL1; NEUROD6; NQO1; NSF; NSG1; NTRK2; PBXIP1; PCP4; PCSK1; PENK; PGAM1; PHGDH; PIP4K2A; PLIN3; PLXNB1; PPAP2B; PRKCB; PTTG1IP; PVALB; RAB31; RASL12; RCAN2; RGS7; RHOBTB3; ROBO3; SCG2; SDC4; SELENBP1; SLC14A1; SLC9A3R1; SMYD2; SOX9; SST; STMN1; STMN2; STS; TAC1; TBL1X; TJAP1; TOB2; TPP1; TSPAN13; TST; TTYH1; TUBB2A; TULP3; TXNIP; WFS1; WSB2; ZHX2 |
| B: *Schizophr Res*. 77:241-52, 2005 (100) | ACTN4; ADCY3; AKAP10; ANKRD10; ANXA1; APOL2; ARRB2; ASCC3; ASH2L; ATM; BCL2L13; BNIP2; BRD1; BRD4; C1S; C2; CAB39L; CARS2; CCDC109B; CCNB1IP1; CDK5RAP1; CIC; CKLF; CLASRP; CLCA4; CMTR1; CNPPD1; COL4A5; CUTC; DFNB31; DHCR7; DNAJC1; DSE; DYRK4; DYSF; EDEM1; ERCC5; FAM49B; FCGR2A; GAA; GADD45A; GBF1; GEMIN6; GLRX; GNE; GOLGA4; GPN3; GPR37; GTF2IRD1; HAPLN2; IFT81; IL1R1; IP6K2; KIAA1324; KLF2; LAMA5; LDOC1; LGALS9; LSM2; LSR; MED15; MGP; MRPS18B; MYL12A; NEMF; NUP37; PCNA; PEX14; PFKFB3; PHC2; PLIN2; PPRC1; QRSL1; RALY; RBMS1; RCL1; REV3L; RING1; RRNAD1; SCAF8; SCARA3; SDPR; SGSH; SLC39A14; SLC44A1; SNRNP40; SORD; STK3; SUSD6; TGIF1; TLR2; TMBIM4; TPST1; TRAF3IP2; TRPC4AP; TTTY15; WDR60; XIST; ZNF175; ZNF189 |
| C: *Schizophr Res*. 161:215-21, 2015 (100) | A2M; ABCG2; ACTR5; AHCYL1; AKAP1; ALDH1L1; ARC; BDKRB1; C17orf80; CASP8; CCL4; CCND2; CDC5L; CFAP70; CHKB; CP; CRH; CRTAM; CSN2; CYTIP; DHRS3; DPYSL3; DUSP1; DUSP5; DUSP6; EGR1; EGR2; EGR4; EPB41L4A-AS2; ETNPPL; ETV5; F7; FBXW11; FGL2; FKBP5; FOS; GDF10; GPR20; GTPBP4; GZMB; HIF3A; HRAS; HSD11B2; HTRA1; IL5; KCNA5; KRT5; KRT6A; MCM6; MDC1; MGEA5; MNS1; MT3; MYBPC1; MYOZ1; NOP14-AS1; NOTCH2NL; NR4A1; NR4A3; OFD1; OSGEP; PAPSS1; PAX4; PDZD2; PLIN1; PRKAB2; PROCR; PTMS; R3HDM2; RASL11B; RERGL; RHOBTB3; RMND5B; RPS17; RPS8; S100A8; SAMHD1; SCGB1D1; SCGB1D2; SCGN; SDC4; SEC14L1; SERPIND1; SLC14A1; SOD3; SPON1; SPRR2C; TEX2; TMPRSS5; TNFSF10; TRIB2; TRIM24; TRPC3; TTC13; UTRN; VEGFC; WNT7A; ZBTB6; ZFP2; ZNF8 |
| D: *Brain Res*. 1239:235-48, 2008 (100) | AARS; ACAT2; ACOX1; ADAM23; ADORA2B; AKR1C3; ALDH4A1; APBB1; APOC1; ARHGEF11; ARL2BP; ATP5A1; BAG3; BBOX1; BEX1; C16orf45; C1R; CCBL2; CCK; CDC42; CHN2; CLUL1; CRHBP; CRYM; CYR61; DDIT4; DHRS3; DNAJC3; DOPEY2; DYNLT3; ELAVL4; ETNPPL; EXTL2; FARP2; FBXO9; FERMT2; GABARAPL1; GAD2; GLUD1; GOLIM4; GOT1; GUCY1B3; HIST1H1C; HSPB1; HTR1F; INHBB; KIAA0101; LAMB2; LAMB3; LEPROT; LRMP; LRPPRC; MAGED1; MAPK9; MBTPS2; MDH1; MPO; MT1X; NDRG3; NEUROD6; NPTX2; NPY; NREP; NSF; OPN3; PALLD; PAM; PARD3; PAX6; PBXIP1; PCP4; PCSK1; PFN2; PMP2; PPME1; PVALB; REEP1; RGS4; SHROOM2; SLC1A1; SLC25A12; SMURF2; SNAP25; SNAP91; SSPN; ST8SIA1; STXBP1; SULF1; SULT4A1; SYNDIG1; TAC1; TGIF1; TPM3; TUBA1B; VIPR1; VSNL1; YAF2; ZC3H13; ZHX2; |

| | |
|---|---|
| | ZNF839 |
| E: *Mol Psychiatry*. 14:1083-94, 2009 (100) | ABCB9; ADAM28; ADAMTS8; ALOX5AP; AMELY; ANP32A-IT1; ARF5; ARID5B; B4GALNT1; BAG3; C11orf80; C1QB; CCDC82; CCND2; CEP131; COL3A1; COL6A2; COLEC10; CRYBG3; CRYM; CTSW; CYP26B1; DFNB31; DKK1; DSG2; DUSP9; EED; FCGR1B; FCGR3B; FGD2; FGF2; FMNL1; FMOD; FRZB; GMPR; GSS; GSTA3; HEBP2; HPCA; HRH3; HSPB1; IKZF5; INSL3; IRF8; JMJD4; JMJD6; KANSL3; KAZALD1; KCTD12; KDM3A; KIAA0408; KREMEN2; LTB; MCM4; MGP; MLNR; MPPED2; MPV17; NEUROD6; NR4A1; NTS; OGN; P4HA1; PEX7; PIEZO1; PLSCR4; PMAIP1; PTK7; PXDN; PYGL; RAPGEF1; RASGRP1; RERGL; RNASET2; S100A12; S100A8; S100A9; SAMSN1; SCIN; SLC13A4; SLC25A24; SLC2A5; SLC30A3; SLCO4A1; SMAD7; SNX6; SYK; TAF12; TCP11; TFDP3; TIMP4; TMEM184C; TOM1; TRAT1; TRIB2; TRPC3; TSR3; UGGT2; ZNF747; ZNRF4 |
| F: *Proc Natl Acad Sci*. 102:15533-8, 2005 (100) | ABCA1; ACO1; ACOX1; ANKRD11; ANKRD12; APC; ARHGEF2; BBX; C1GALT1; C1orf216; C21orf59; CADM1; CCS; CEP290; CHRDL1; CNTNAP1; COL16A1; CORO7; CYP20A1; DCAF11; DIRAS2; DNAAF5; DUSP14; DYNC1LI2; ENAH; ERBB4; FABP6; FAIM2; FAP; FBXL14; FGFR3; FNBP1L; FNDC3B; GABARAPL1; GABRA2; GABRB1; GANAB; GRAMD1C; GSK3A; HIRA; HMGN1; HSP90B1; IFT140; IGF1R; ITGA8; KBTBD4; KIF5B; KLHDC3; LPL; LSG1; LUC7L3; MARC2; MRPS16; MSL1; NKTR; NOL3; NR2E1; NTM; NTSR2; PCK2; PCSK5; PHF21A; PIP4K2C; PLCXD1; PLOD2; POU3F2; PPAP2B; PPIG; PRPF19; PRRC2C; RABGAP1; RBL2; RETSAT; RGS20; RPL27A; RYR3; SCAMP5; SCAPER; SCRG1; SEC61B; SFXN3; SLC12A7; SLC1A2; SMIM14; SOX9; SRI; SRRD; SSBP3; SSPN; SUPT5H; TBC1D10B; TESPA1; TMEM47; TMEM50A; TNIK; TPBG; UXS1; VEGFA; WARS2; ZBBX |
| G: *PLoS One*. 10:e0121744, 2015 (100) | ADAM11; ADRBK2; ALDH7A1; ANGPTL4; APOD; ATP1B2; ATP6V0A1; ATP6V1A; BAG3; BCL6; BDH2; CASP7; CD81; CEP76; CHRDL1; CIDEA; CNTNAP2; DECR1; DNAJC28; EDN3; ELOVL5; EPN3; ERLIN2; ETNPPL; F3; FABP3; FAM65B; FGF2; FOLR1; GFRA2; GNB5; GNG12; GPC5; GTF2F1; HADHB; HMGN1; HS3ST1; HS3ST3B1; HSDL2; IFITM2; IFITM3; IGFBP6; IL17RB; ISLR; ITGAV; KCNS3; KIF5A; KLF15; KLF5; LONRF3; MARK2; MEIS2; METTL7A; MREG; MT2A; MT3; MTM1; MTUS2; NDRG4; NOTCH2; OCEL1; OSTF1; PAAF1; PIGA; PIK3CB; PLXDC1; POLR2E; PRKCI; PRSS2; PTPN13; PTPRZ1; PVALB; RABEP2; RFX4; RGS6; RRN3; SCN1B; SDC4; SIRT1; SLC19A2; SLC6A16; SLC7A11; SLCO1C1; SMAD1; SNX15; SOX2; SPON1; SUCO; SYNDIG1; TOLLIP; TRAK2; TUBB2B; UNG; USP3; WIF1; WIPI1; XPO1; ZC3H13; ZMAT4; ZNF267 |
| H: *BMC Psychiatry*. 8:87, 2008 (100) | ACTR6; AGL; ALG5; ARMT1; ATG3; ATP1A3; ATP5G1; AVL9; BDNF; BET1; CBX1; CCDC25; CHMP5; CLNS1A; DCUN1D1; DENR; DGUOK; DIABLO; DROSHA; EDN1; EIF4G1; ENOPH1; ERCC5; FADS3; GGT5; GNPDA1; GRHPR; GSPT2; GTF2B; HEY1; HIBCH; IFT52; IL4R; KIAA0232; KIAA0368; KIAA0895; KIAA0930; LACTB2; LRPPRC; LRRC49; LYRM4; MAGEF1; MAPK6; MED4; MED7; METTL18; MRPL48; MXD3; NDUFB1; NOC3L; NUP58; OARD1; OPA1; OSBPL2; PDCD10; PIN4; PLA1A; PLA2G16; POLB; PPP1CC; PPP3CB; PPT1; PSMA7; PSMB4; PSMD4; PTBP2; QDPR; RBM15; RCAN2; RHBDF1; RPA1; RPAP3; SEPHS1; SGMS1; SHFM1; SNRPB2; SNRPE; SNX19; STAM; STK39; SUSD5; TAF7; TCFL5; THOC7; TMEM176A; TOB1; TSN; TTC1; TXN; TXNDC9; UBE2O; UPF3B; VAMP2; VPS26A; VPS41; ZBTB24; ZCCHC10; ZNHIT3; ZYX; ZZZ3 |
| I: *Neuropsychopharmacol H*. 10:9-14, 2008 | A2M; ABCG2; ACOT13; ACTA1; ADAM28; ADCYAP1; ADI1; AGL; APOA4; ARMC6; ARMCX5; ATP10D; BCL6; BLNK; C3; CBS; CCDC181; CCL25; CD37; CDADC1; COPS5; CST1; CTH; CXCL14; CXorf36; CYBB; CYTL1; |

| | |
|---|---|
| (100) | DAO; DCP2; DUSP5; ERC1; FAM35A; FAM64A; GMNN; GNA14; GNE; GOLGA2P5; GPR182; GSTO1; HARS2; HLA-DQA1; HMGB2; HOXD9; LARP4; LGALS2; LPL; MAFF; MNS1; MORN1; MTMR12; NCF2; NEDD9; NFIL3; NLGN4X; NUDT7; NUP98; P2RX4; PCSK1; PIKFYVE; PLEKHF2; PLOD2; PROC; RAB8A; RAP2C; RBM4B; RCOR1; RSPH6A; SCAF8; SCML1; SDC4; SELPLG; SESN1; SIX1; SLC12A2; SLC25A24; SLC30A10; SLCO2B1; SOCS7; SUCO; SYNPO; TBXAS1; TM6SF2; TNFSF10; TOMM40; TTC17; TTC30A; TUBGCP4; TVP23B; USP3; VCX2; WBP1L; WDR19; WDR62; WIPI1; XAB2; ZBED8; ZIC3; ZNF606; ZNF75D; ZWINT |

**Supplementary Table S5.** The reproducibility of two popular feature selection methods (Student's *t*-test and SAM) and the new strategy proposed by this study. The reproducibility was assessed using the accuracy (ACC) & the Matthews correlation coefficient (MCC) of each study listed in **Table 1** on the remaining eight datasets (**Table 1**).

| Training Datasets | Test Datasets | Measure | This Study | Student's *t*-test | SAM |
|---|---|---|---|---|---|
| **Study B**: *Schizophr Res.* 77:241-52, 2005 | A: *BMC Genomics*. 7:70, 2006 | ACC (%) | 75.9 | 53.8 | 50.8 |
| | | MCC | 0.52 | 0.20 | 0.17 |
| | C: *Schizophr Res*. 161:215-21, 2015 | ACC (%) | 57.6 | 55.9 | 61.0 |
| | | MCC | 0.20 | 0.15 | 0.23 |
| | D: *Brain Res*. 1239:235-48, 2008 | ACC (%) | 77.8 | 57.4 | 59.3 |
| | | MCC | 0.50 | 0.21 | 0.19 |
| | E: *Mol Psychiatry*. 14:1083-94, 2009 | ACC (%) | 70.2 | 66.0 | 63.8 |
| | | MCC | 0.41 | 0.31 | 0.27 |
| | F: *Proc Natl Acad Sci U S A*. 102:15533-8, 2005 | ACC (%) | 68.9 | 60.0 | 60.0 |
| | | MCC | 0.38 | 0.18 | 0.18 |
| | G: *PLoS One*. 10:e0121744, 2015 | ACC (%) | 78.1 | 56.3 | 59.4 |
| | | MCC | 0.55 | -0.01 | 0.05 |
| | H: *BMC Psychiatry*. 8:87, 2008 | ACC (%) | 60.0 | 60.0 | 60.0 |
| | | MCC | 0.25 | 0.25 | 0.18 |
| | I: *Neuropsychopharmacol H*. 10:9-14, 2008 | ACC (%) | 73.3 | 66.7 | 66.7 |
| | | MCC | 0.48 | 0.29 | 0.29 |
| **Study C**: *Schizophr Res*. 161:215-21, 2015 | A: *BMC Genomics*. 7:70, 2006 | ACC (%) | 84.6 | 67.7 | 64.6 |
| | | MCC | 0.70 | 0.37 | 0.34 |
| | B: *Schizophr Res*. 77:241-52, 2005 | ACC (%) | 68.3 | 56.7 | 61.7 |
| | | MCC | 0.38 | 0.13 | 0.23 |
| | D: *Brain Res*. 1239:235-48, 2008 | ACC (%) | 66.7 | 59.3 | 61.1 |
| | | MCC | 0.34 | 0.26 | 0.21 |
| | E: *Mol Psychiatry*. 14:1083-94, 2009 | ACC (%) | 74.5 | 63.8 | 68.1 |
| | | MCC | 0.51 | 0.34 | 0.39 |
| | F: *Proc Natl Acad Sci U S A*. 102:15533-8, 2005 | ACC (%) | 64.4 | 68.9 | 64.4 |
| | | MCC | 0.31 | 0.35 | 0.28 |
| | G: *PLoS One*. 10:e0121744, 2015 | ACC (%) | 84.4 | 68.8 | 68.8 |
| | | MCC | 0.67 | 0.35 | 0.34 |

| | | | | | |
|---|---|---|---|---|---|
| | H: *BMC Psychiatry*. 8:87, 2008 | ACC (%) | 80.0 | 65.0 | 65.0 |
| | | MCC | 0.62 | 0.37 | 0.30 |
| | I: *Neuropsychopharmacol H*. 10:9-14, 2008 | ACC (%) | 66.7 | 60.0 | 66.7 |
| | | MCC | 0.33 | 0.41 | 0.39 |
| **Study D**: *Brain Res*. 1239:235-48, 2008 | A: *BMC Genomics*. 7:70, 2006 | ACC (%) | 75.4 | 60.0 | 61.5 |
| | | MCC | 0.51 | 0.20 | 0.26 |
| | B: *Schizophr Res*. 77:241-52, 2005 | ACC (%) | 71.7 | 63.3 | 61.7 |
| | | MCC | 0.51 | 0.27 | 0.25 |
| | C: *Schizophr Res*. 161:215-21, 2015 | ACC (%) | 69.5 | 52.5 | 66.1 |
| | | MCC | 0.42 | 0.13 | 0.34 |
| | E: *Mol Psychiatry*. 14:1083-94, 2009 | ACC (%) | 70.2 | 57.4 | 63.8 |
| | | MCC | 0.47 | 0.11 | 0.27 |
| | F: *Proc Natl Acad Sci U S A*. 102:15533-8, 2005 | ACC (%) | 68.9 | 64.4 | 64.4 |
| | | MCC | 0.36 | 0.25 | 0.25 |
| | G: *PLoS One*. 10:e0121744, 2015 | ACC (%) | 71.9 | 65.6 | 62.5 |
| | | MCC | 0.46 | 0.26 | 0.22 |
| | H: *BMC Psychiatry*. 8:87, 2008 | ACC (%) | 75.0 | 60.0 | 60.0 |
| | | MCC | 0.50 | 0.21 | 0.19 |
| | I: *Neuropsychopharmacol H*. 10:9-14, 2008 | ACC (%) | 73.3 | 66.7 | 60.0 |
| | | MCC | 0.58 | 0.29 | 0.29 |
| **Study E**: *Mol Psychiatry*. 14:1083-94, 2009 | A: *BMC Genomics*. 7:70, 2006 | ACC (%) | 68.9 | 67.7 | 69.2 |
| | | MCC | 0.36 | 0.35 | 0.40 |
| | B: *Schizophr Res*. 77:241-52, 2005 | ACC (%) | 63.3 | 58.3 | 50.0 |
| | | MCC | 0.32 | 0.16 | 0.13 |
| | C: *Schizophr Res*. 161:215-21, 2015 | ACC (%) | 66.1 | 55.9 | 64.4 |
| | | MCC | 0.34 | 0.12 | 0.29 |
| | D: *Brain Res*. 1239:235-48, 2008 | ACC (%) | 79.6 | 70.4 | 64.8 |
| | | MCC | 0.60 | 0.45 | 0.32 |
| | F: *Proc Natl Acad Sci U S A*. 102:15533-8, 2005 | ACC (%) | 68.9 | 64.4 | 64.4 |
| | | MCC | 0.36 | 0.27 | 0.31 |
| | G: *PLoS One*. 10:e0121744, 2015 | ACC (%) | 78.1 | 68.8 | 68.8 |
| | | MCC | 0.51 | 0.33 | 0.46 |
| | H: *BMC Psychiatry*. 8:87, 2008 | ACC (%) | 68.9 | 65.0 | 60.0 |
| | | MCC | 0.36 | 0.37 | 0.25 |

| | | | | | |
|---|---|---|---|---|---|
| | I: *Neuropsychopharmacol H.* 10:9-14, 2008 | ACC (%) | 75.5 | 66.7 | 66.7 |
| | | MCC | 0.55 | 0.33 | 0.27 |
| **Study F**: *Proc Natl Acad Sci U S A*. 102:15533-8, 2005 | A: *BMC Genomics*. 7:70, 2006 | ACC (%) | 70.8 | 67.7 | 63.1 |
| | | MCC | 0.41 | 0.35 | 0.27 |
| | B: *Schizophr Res*. 77:241-52, 2005 | ACC (%) | 80.0 | 70.0 | 70.0 |
| | | MCC | 0.61 | 0.44 | 0.43 |
| | C: *Schizophr Res*. 161:215-21, 2015 | ACC (%) | 69.5 | 64.4 | 64.4 |
| | | MCC | 0.41 | 0.31 | 0.29 |
| | D: *Brain Res*. 1239:235-48, 2008 | ACC (%) | 72.2 | 63.0 | 66.7 |
| | | MCC | 0.44 | 0.27 | 0.34 |
| | E: *Mol Psychiatry*. 14:1083-94, 2009 | ACC (%) | 66.0 | 55.3 | 66.0 |
| | | MCC | 0.36 | 0.19 | 0.31 |
| | G: *PLoS One*. 10:e0121744, 2015 | ACC (%) | 84.4 | 68.8 | 71.9 |
| | | MCC | 0.67 | 0.33 | 0.51 |
| | H: *BMC Psychiatry*. 8:87, 2008 | ACC (%) | 75.0 | 65.0 | 55.0 |
| | | MCC | 0.55 | 0.37 | 0.30 |
| | I: *Neuropsychopharmacol H.* 10:9-14, 2008 | ACC (%) | 80.0 | 66.7 | 73.3 |
| | | MCC | 0.60 | 0.39 | 0.48 |
| **Study G**: *PLoS One*. 10:e0121744, 2015 | A: *BMC Genomics*. 7:70, 2006 | ACC (%) | 76.9 | 61.5 | 66.2 |
| | | MCC | 0.55 | 0.23 | 0.38 |
| | B: *Schizophr Res*. 77:241-52, 2005 | ACC (%) | 61.7 | 51.7 | 58.3 |
| | | MCC | 0.30 | 0.07 | 0.23 |
| | C: *Schizophr Res*. 161:215-21, 2015 | ACC (%) | 62.7 | 69.5 | 69.5 |
| | | MCC | 0.29 | 0.42 | 0.40 |
| | D: *Brain Res*. 1239:235-48, 2008 | ACC (%) | 75.9 | 70.4 | 74.1 |
| | | MCC | 0.52 | 0.43 | 0.54 |
| | E: *Mol Psychiatry*. 14:1083-94, 2009 | ACC (%) | 76.6 | 68.1 | 68.1 |
| | | MCC | 0.53 | 0.35 | 0.35 |
| | F: *Proc Natl Acad Sci U S A*. 102:15533-8, 2005 | ACC (%) | 68.9 | 66.7 | 68.9 |
| | | MCC | 0.38 | 0.37 | 0.41 |
| | H: *BMC Psychiatry*. 8:87, 2008 | ACC (%) | 75.0 | 65.0 | 70.0 |
| | | MCC | 0.53 | 0.37 | 0.41 |
| | I: *Neuropsychopharmacol H.* 10:9-14, 2008 | ACC (%) | 80.0 | 66.7 | 73.3 |
| | | MCC | 0.61 | 0.49 | 0.58 |

| | | | | | |
|---|---|---|---|---|---|
| **Study H**: *BMC Psychiatry*. 8:87, 2008 | A: *BMC Genomics*. 7:70, 2006 | ACC (%) | 66.2 | 56.9 | 60.0 |
| | | MCC | 0.35 | 0.30 | 0.20 |
| | B: *Schizophr Res*. 77:241-52, 2005 | ACC (%) | 63.3 | 65.0 | 55.0 |
| | | MCC | 0.34 | 0.32 | 0.21 |
| | C: *Schizophr Res*. 161:215-21, 2015 | ACC (%) | 55.9 | 55.9 | 55.9 |
| | | MCC | 0.16 | 0.13 | 0.12 |
| | D: *Brain Res*. 1239:235-48, 2008 | ACC (%) | 64.8 | 57.4 | 57.4 |
| | | MCC | 0.29 | 0.14 | 0.13 |
| | E: *Mol Psychiatry*. 14:1083-94, 2009 | ACC (%) | 76.6 | 70.2 | 66.0 |
| | | MCC | 0.54 | 0.41 | 0.31 |
| | F: *Proc Natl Acad Sci U S A*. 102:15533-8, 2005 | ACC (%) | 66.7 | 62.2 | 64.4 |
| | | MCC | 0.33 | 0.25 | 0.33 |
| | G: *PLoS One*. 10:e0121744, 2015 | ACC (%) | 65.6 | 62.5 | 62.5 |
| | | MCC | 0.26 | 0.22 | 0.22 |
| | I: *Neuropsychopharmacol H*. 10:9-14, 2008 | ACC (%) | 73.3 | 53.3 | 66.7 |
| | | MCC | 0.58 | 0.32 | 0.39 |
| **Study I**: *Neuropsychopharmacol H*. 10:9-14, 2008 | A: *BMC Genomics*. 7:70, 2006 | ACC (%) | 66.2 | 61.5 | 61.5 |
| | | MCC | 0.34 | 0.24 | 0.25 |
| | B: *Schizophr Res*. 77:241-52, 2005 | ACC (%) | 58.3 | 61.7 | 60.0 |
| | | MCC | 0.17 | 0.24 | 0.31 |
| | C: *Schizophr Res*. 161:215-21, 2015 | ACC (%) | 66.1 | 62.7 | 72.9 |
| | | MCC | 0.36 | 0.26 | 0.47 |
| | D: *Brain Res*. 1239:235-48, 2008 | ACC (%) | 68.5 | 64.8 | 55.6 |
| | | MCC | 0.42 | 0.30 | 0.15 |
| | E: *Mol Psychiatry*. 14:1083-94, 2009 | ACC (%) | 66.0 | 63.8 | 60.0 |
| | | MCC | 0.31 | 0.29 | 0.19 |
| | F: *Proc Natl Acad Sci U S A*. 102:15533-8, 2005 | ACC (%) | 68.9 | 64.4 | 64.4 |
| | | MCC | 0.40 | 0.25 | 0.25 |
| | G: *PLoS One*. 10:e0121744, 2015 | ACC (%) | 78.1 | 65.6 | 65.6 |
| | | MCC | 0.55 | 0.27 | 0.26 |
| | H: *BMC Psychiatry*. 8:87, 2008 | ACC (%) | 65.0 | 60.0 | 60.0 |
| | | MCC | 0.37 | 0.38 | 0.38 |

**Supplementary Table S6.** 33 DEGs discovered by at least 3 out of 6 datasets with large sample size (>40) using the new strategy proposed in this study (ordered by the gene symbols of 33 DEGs).

| Label | Gene Symbol | Protein Name | Entrez ID | Probe ID |
|---|---|---|---|---|
| DEG01 | ADM | Adrenomedullin | 133 | 218322_s_at |
| DEG02 | ALB | Serum albumin | 213 | 202834_at |
| DEG03 | ANKRD1 | Ankyrin repeat domain-containing protein 1 | 27063 | 217626_at |
| DEG04 | ARMCX5 | Armadillo repeat-containing X-linked 5 | 64860 | 218489_s_at |
| DEG05 | CCBL2 | Kynurenine-oxoglutarate transaminase 3 | 56267 | 208220_x_at |
| DEG06 | CNTRL | Centriolin | 11064 | 202204_s_at |
| DEG07 | CP | Ceruloplasmin | 1356 | 212747_at |
| DEG08 | CRB1 | Protein crumbs homolog 1 | 23418 | 211047_x_at |
| DEG09 | DGCR9 | (non-protein coding) | 25787 | 219335_at |
| DEG10 | FJX1 | Four-jointed box protein 1 | 24147 | 203388_at |
| DEG11 | GSC2 | Homeobox protein goosecoid-2 | 2928 | 213902_at |
| DEG12 | HOXB9 | Homeobox protein Hox-B9 | 3219 | 208833_s_at |
| DEG13 | HSD11B1 | 11-beta-hydroxysteroid dehydrogenase 1 | 3290 | 217925_s_at |
| DEG14 | KCNA3 | Voltage-gated K(+) channel HuKIII | 3738 | 210108_at |
| DEG15 | KDM5D | Lysine-specific demethylase 5D | 8284 | 203538_at |
| DEG16 | MEFV | Marenostrin | 4210 | 207962_at |
| DEG17 | MFSD1 | Smooth muscle cell-associated protein 4 | 64747 | 209472_at |
| DEG18 | MGP | Matrix Gla protein | 4256 | 203119_at |
| DEG19 | MICAL2 | Molecule interacting with CasL protein 2 | 9645 | 214230_at |
| DEG20 | NXF1 | Nuclear RNA export factor 1 | 10482 | 214797_s_at |
| DEG21 | PCSK6 | Proprotein convertase subtilisin/kexin 6 | 5046 | 201445_at |
| DEG22 | PEX14 | Peroxisomal membrane protein PEX14 | 5195 | 211966_at |
| DEG23 | PLCG1 | Phosphoinositide phospholipase C-gamma-1 | 5335 | 220522_at |
| DEG24 | RAPGEF1 | Rap guanine nucleotide exchange factor 1 | 2889 | 218097_s_at |
| DEG25 | RGN | Regucalcin | 9104 | 214112_s_at |
| DEG26 | RPL36 | 60S ribosomal protein L36 | 25873 | 219825_at |
| DEG27 | S100A8 | Protein S100-A8 | 6279 | 210764_s_at |
| DEG28 | SCN1B | Sodium channel subunit beta-1 | 6324 | 219945_at |
| DEG29 | SOX30 | Transcription factor SOX-30 | 11063 | 202447_at |
| DEG30 | TAC1 | Protachykinin-1 | 6863 | 203694_s_at |
| DEG31 | TNFSF10 | TNF-related apoptosis-inducing ligand | 8743 | 205744_at |
| DEG32 | TRAT1 | T-cell receptor-interacting molecule | 50852 | 221817_at |
| DEG33 | TXNL4B | Thioredoxin-like protein 4B | 54957 | 209950_s_at |

**Supplementary Table S7.** The relevance between each DEG identified by this study and psychological mechanisms underlining SCZ's cognitive dysfunction confirmed by published literatures. 25 (75.8%) out of those 33 DEGs discovered by this study were found to be closely related.

| Gene Symbol | Relevance between SCZ and the Identified DEGs Confirmed by the Comprehensive Literature Reviews |
|---|---|
| ADM | A promising biomarker of SCZ and up-regulated in both the LB cells and plasma of SCZ patients[1]. Its expression was significantly altered during pre-dementia stage of mild cognitive impairment[2], and it prevented cognitive decline after chronic cerebral hypoperfusion[3]. |
| ALB | The expression level of serum albumin (ALB) was down-regulated in SCZ patients[4], and it was differentially expressed in the plasma of mild cognitive impaired subjects[5]. |
| ANKRD1 | The expression of ANKRD1 was upregulated by ZNF804A which was a candidate risk gene for SCZ and affected the cognitive functions including verbal and spatial working memory[6]. |
| ARMCX5 | ARMCX5 interacted with GTF2IRD1 which was considered as the childhood-onset SCZ candidate gene[7]. |
| CCBL2 | CCBL2 catalyzed the central and peripheral formation of kynurenic acid (KYNA)[8], which was associated with the cognitive impairments in SCZ[9]. |
| CP | Increases in ceruloplasmin (CP) may result in increased levels of copper, which ultimately proves deleterious in SCZ[10]. Novel mutation in ceruloplasmin gene causes a cognitive and movement disorder[11]. |
| CRB1 | CRB1 was differentially expressed between SCZ patients and healthy controls[12]. |
| GSC2 | GSC2 are encompassed in SCZ-associated deleted region (22q11.2) and patients with 22q11.2 deletion syndrome are associated with a high rate (25%) of SCZ[13]. |
| HOXB9 | HOXB9 was downregulated by miRNA microarray analysis in the target analysis of SCZ associated microRNAs[14]. |
| HSD11B1 | The HSD11B1 gene encoding proteins associated with lipid metabolic processes presented a different expression in SCZ patients compared to controls[15]. |
| KCNA3 | KCNA3 was regulated by KCNE2 gene, the sequence variants or duplications of which was associated with SCZ[16]. |
| KDM5D | KDM5D was sex-associated differences in ADNP+/+ and ADNP+/−, and ADNP is sexually regulated in lymphocytes of SCZ patients[17]. |
| MEFV | MEFV mutation may have a protective effect on cognitive impairment with unknown mechanism[18]. |
| MFSD1 | Hypoxia has been identified as a strong risk factor in SCZ, but MFSD1 involved in cell stabilization was upregulated, which may reflect compensatory responses[19]. |

| | |
|---|---|
| NXF1 | NXF1-associated gene expression and protein networks that interact with miRNAs was found in cognitive impairment and developmental cognitive disorder[20]. |
| PCSK6 | The PCSK6 VNTR genotypes mediate the expression of psychological phenotypes that involve atypical cerebral lateralization, such that this locus apparently exerts pleiotropic effects on both handedness and psychological-cognitive phenotypes[21]. |
| PEX14 | PEX14 was down-regulated in SZ throughout different brain regions[22]. The patient began to have symptoms of cognitive deterioration at 9 years of age, presented a mutation in the PEX14 gene[23]. |
| PLCG1 | PLCG1 was found in high frequency in the top ranked signaling pathways, which were known to be of importance in SCZ[24]. The abnormal expression and activation of PLCG1 resulted in devastating cognitive, psychological and motor disturbances[25]. |
| RAPGEF1 | Using qPCR, we confirmed that levels of mRNA for RAPGEF1 ($P<0.05$) was lower in the SCZ comparing controls[26]. |
| RGN | Interestingly, the concentration of regucalcin (RGN) in cerebral cortex and hippocampus is decreased with aging, and the changes in the neuronal $Ca^{2+}$ homeostasis with aging may be implicated in age-related disturbance in cognitive functions[27]. |
| RPL36 | RPL36 is downregulated in validated target genes between SCZ and controls in the whole blood microRNA levels[28]. |
| S100A8 | S100A8 consistently changed in expression between schizophrenic patients and controls and were nominally significant in the gene-based association analysis[29], and it contributed to postoperative cognitive dysfunction in mice undergoing tibial fracture surgery[30]. |
| SCN1B | A dysregulated gene in SCZ patients[31]. The homozygous SCN1B mutations indicated that SCN1B was an etiologic candidate underlying *dravet syndrome* which was characterized by early onset epileptic seizures followed by ataxia and cognitive decline[32]. |
| TAC1 | The trend for TAC1 was for decreased density in SCZ patients[33]. It emerged as a top candidate gene for cognitive disorders in a unique multi-stage analysis of human genetic linkage[34,35]. |
| TNFSF10 | TNFSF10 was consistently found differentially expressed between SCZ subjects and healthy controls[36]. An anti-TNFSF10 antibody could reduce brain amyloid-β load and activation of TNFSF10 apoptotic receptors, as well as improve cognition[37]. |

**References for Table S7:**


1 C. Kakiuchi; M. Ishiwata; S. Nanko; N. Ozaki; N. Iwata; T. Umekage; M. Tochigi; K. Kohda; T. Sasaki; A. Imamura; Y. Okazaki; T. Kato. Up-regulation of ADM and SEPX1 in the lymphoblastoid cells of patients in monozygotic twins discordant for schizophrenia. Am J Med Genet B Neuropsychiatr Genet. 2008, 147B(5): 557-64

2 A. P. Fernandez; J. S. Masa; M. A. Guedan; H. S. Futch; R. Martinez-Murillo. Adrenomedullin Expression in Alzheimer's Brain. Curr Alzheimer Res. 2016, 13(4): 428-38

3 T. Maki; M. Ihara; Y. Fujita; T. Nambu; K. Miyashita; M. Yamada; K. Washida; K. Nishio; H. Ito; H. Harada; H. Yokoi; H. Arai; H. Itoh; K. Nakao; R. Takahashi; H. Tomimoto. Angiogenic and vasoprotective effects of adrenomedullin on prevention of cognitive decline after chronic cerebral hypoperfusion in mice. Stroke. 2011, 42(4): 1122-8

4 D. Zhai; Y. Liu; F. Ma; Y. Feng; Z. Xu; T. Cui; Y. Lang; X. Wang; Y. Cao; Y. Zhao; R. Zhang; X. Zhang. Effects of the First Exposure of Antipsychotics on Serum Albumin in Adolescents and Young Adults With First-Episode Schizophrenia. J Clin Psychopharmacol. 2018, 38(1): 103-05

5 A. Kumar; S. Singh; A. Verma; V. N. Mishra. Proteomics based identification of differential plasma proteins and changes in white matter integrity as markers in early detection of mild cognitive impaired subjects at high risk of Alzheimer's disease. Neurosci Lett. 2018, 67671-77

6 J. L. Hess; S. J. Glatt. How might ZNF804A variants influence risk for schizophrenia and bipolar disorder? A literature review, synthesis, and bioinformatic analysis. Am J Med Genet B Neuropsychiatr Genet. 2014, 165B(1): 28-40

7 A. Ambalavanan; S. L. Girard; K. Ahn; S. Zhou; A. Dionne-Laporte; D. Spiegelman; C. V. Bourassa; J. Gauthier; F. F. Hamdan; L. Xiong; P. A. Dion; R. Joober; J. Rapoport; G. A. Rouleau. De novo variants in sporadic cases of childhood onset schizophrenia. Eur J Hum Genet. 2016, 24(6): 944-8

8 A. S. Johansson; B. Owe-Larsson; L. Asp; T. Kocki; M. Adler; J. Hetta; R. Gardner; G. B. Lundkvist; E. M. Urbanska; H. Karlsson. Activation of kynurenine pathway in ex vivo fibroblasts from patients with bipolar disorder or schizophrenia: cytokine challenge increases production of 3-hydroxykynurenine. J Psychiatr Res. 2013, 47(11): 1815-23

9 M. M. Koola. Kynurenine pathway and cognitive impairments in schizophrenia: Pharmacogenetics of galantamine and memantine. Schizophr Res Cogn. 2016, 44-9

10 A. Czinner; M. Neuwirth. Thrombosis of the anterior cerebral artery related to primary hyperlipoproteinemia, type IIa in childhood. Kinderarztl Prax. 1983, 51(1): 32-6

11 H. F. Shang; X. F. Jiang; J. M. Burgunder; Q. Chen; D. Zhou. Novel mutation in the ceruloplasmin gene causing a cognitive and movement disorder with diabetes mellitus. Mov Disord. 2006, 21(12): 2217-20

12 M. Mistry; J. Gillis; P. Pavlidis. Genome-wide expression profiling of schizophrenia using a large combined cohort. Mol Psychiatry. 2013, 18(2): 215-25

13 C. P. Chen; J. P. Huang; Y. Y. Chen; S. R. Chern; P. S. Wu; J. W. Su; Y. T. Chen; W. L. Chen; W. Wang. Chromosome 22q11.2 deletion syndrome: prenatal diagnosis, array comparative genomic hybridization characterization using uncultured amniocytes and literature review. Gene. 2013, 527(1): 405-9

14 T. I. Vachev; N. T. Popov; D. Marchev; H. Ivanov; V. K. Stoyanova. Characterization of Micro RNA Signature in Peripheral Blood of Schizophrenia Patients using µParaflo™ miRNA Microarray Assay. Int J Curr Microbiol App Sci. 2016, 5(7): 503-12

15 M. Logotheti; O. Papadodima; N. Venizelos; A. Chatziioannou; F. Kolisis. A comparative genomic study in schizophrenic and in bipolar disorder patients, based on microarray expression profiling meta-analysis. ScientificWorldJournal. 2013, 2013685917

16 T. K. Roepke; V. A. Kanda; K. Purtell; E. C. King; D. J. Lerner; G. W. Abbott. KCNE2 forms potassium channels with KCNA3 and KCNQ1 in the choroid plexus epithelium. FASEB J. 2011, 25(12): 4264-73

17 I. Gozes. Sexual divergence in activity-dependent neuroprotective protein impacting autism, schizophrenia, and Alzheimer's disease. J Neurosci Res. 2017, 95(1-2): 652-60

18 G. Keskindemirci; G. Eskikurt; N. A. Ayaz; M. Cakan; N. Ermutlu; U. Isoglu Alkac. Does familial Mediterranean fever affect cognitive function in children? Electrophysiological preliminary study. Int J Neurosci. 2018, 128(1): 10-14

19 C. Sellmann; L. Villarin Pildain; A. Schmitt; F. Leonardi-Essmann; P. F. Durrenberger; R. Spanagel; T. Arzberger; H. Kretzschmar; M. Zink; O. Gruber; M. Herrera-Marschitz; R. Reynolds; P. Falkai; P. J. Gebicke-Haerter; F. Matthaus. Gene expression in superior temporal cortex of schizophrenia patients. Eur Arch Psychiatry Clin Neurosci. 2014, 264(4): 297-309

20 P. Shapshak. Molecule of the month: miRNA and Down's syndrome. Bioinformation. 2013, 9(15): 752-4

21 K. J. Robinson; P. L. Hurd; S. Read; B. J. Crespi. The PCSK6 gene is associated with handedness, the autism spectrum, and magical ideation in a non-clinical population. Neuropsychologia. 2016, 84205-12

22 Y. Horesh; P. Katsel; V. Haroutunian; E. Domany. Gene expression signature is shared by patients with Alzheimer's disease and schizophrenia at the superior temporal gyrus. Eur J Neurol. 2011, 18(3): 410-24

23 A. Galvez-Ruiz; A. Galindo-Ferreiro; H. Alkatan. A clinical case of Zellweger syndrome in a patient with a previous history of ocular medulloepithelioma. Saudi J Ophthalmol. 2017, doi: 10.1016/j.sjopt.2017.09.004

24 S. Chandrasekaran; D. G. Bonchev. A network view on Schizophrenia related genes. Network Biology. 2012, 2(1): 16-25

25 A. Giralt; T. Rodrigo; E. D. Martin; J. R. Gonzalez; M. Mila; V. Cena; M. Dierssen; J. M. Canals; J. Alberch. Brain-derived neurotrophic factor modulates the severity of cognitive alterations induced by mutant huntingtin: involvement of phospholipaseCgamma activity and glutamate receptor expression. Neuroscience. 2009, 158(4): 1234-50

26 E. Scarr; M. Udawela; E. A. Thomas; B. Dean. Changed gene expression in subjects with schizophrenia and low cortical muscarinic M1 receptors predicts disrupted upstream pathways interacting with that receptor. Mol Psychiatry. 2018, 23(2): 295-303

27 M. Yamaguchi. Regucalcin and cell regulation: role as a suppressor protein in signal transduction. Mol Cell Biochem. 2011, 353(1-2): 101-37

28 T. I. Vachev; N. T. Popov; V. K. Stoyanova; H. Y. Ivanov; D. S. Minchev. Down Regulation of MIR-320 Gene Family Members in the Peripheral Blood of Schizophrenia Patients. Int J Curr Microbiol App Sci. 2016, 5(1): 221-30

29 J. Xu; J. Sun; J. Chen; L. Wang; A. Li; M. Helm; S. L. Dubovsky; S. A. Bacanu; Z. Zhao; X. Chen. RNA-Seq analysis implicates dysregulation of the immune system in schizophrenia. BMC Genomics. 2012, 13 Suppl 8S2

30 S. M. Lu; C. J. Yu; Y. H. Liu; H. Q. Dong; X. Zhang; S. S. Zhang; L. Q. Hu; F. Zhang; Y. N. Qian; B. Gui. S100A8 contributes to postoperative cognitive dysfunction in mice undergoing tibial fracture surgery by activating the TLR4/MyD88 pathway. Brain Behav Immun. 2015, 44221-34

31 J. Perez-Santiago; R. Diez-Alarcia; L. F. Callado; J. X. Zhang; G. Chana; C. H. White; S. J. Glatt; M. T. Tsuang; I. P. Everall; J. J. Meana; C. H. Woelk. A combined analysis of microarray gene expression studies of the human prefrontal cortex identifies genes implicated in schizophrenia. J Psychiatr Res. 2012, 46(11): 1464-74

32 I. Ogiwara; T. Nakayama; T. Yamagata; H. Ohtani; E. Mazaki; S. Tsuchiya; Y. Inoue; K. Yamakawa. A homozygous mutation of voltage-gated sodium channel beta(I) gene SCN1B in a patient with Dravet syndrome. Epilepsia. 2012, 53(12): e200-3

33 A. L. Guillozet-Bongaarts; T. M. Hyde; R. A. Dalley; M. J. Hawrylycz; A. Henry; P. R. Hof; J. Hohmann; A. R. Jones; C. L. Kuan; J. Royall; E. Shen; B. Swanson; H. Zeng; J. E. Kleinman. Altered gene expression in the dorsolateral prefrontal cortex of individuals with schizophrenia. Mol Psychiatry. 2014, 19(4): 478-85

34 J. Fossella; J. Fan; X. Liu; K. Guise; K. Brocki; P. R. Hof; R. Kittappa; R. McKay; M. Posner. Provisional hypotheses for the molecular genetics of cognitive development: imaging genetic pathways in the anterior cingulate cortex. Biol Psychol. 2008, 79(1): 23-9

35 E. Bora; B. J. Harrison; M. Yucel; C. Pantelis. Cognitive impairment in euthymic major depressive disorder: a meta-analysis. Psychol Med. 2013, 43(10): 2017-26

36 J. Struyf; S. Dobrin; D. Page. Combining gene expression, demographic and clinical data in modeling disease: a case study of bipolar disorder and schizophrenia. BMC Genomics. 2008, 9531

37 D. Frenkel. A new TRAIL in Alzheimer's disease therapy. Brain. 2015, 138(Pt 1): 8-10

**Supplementary Table S8.** The top-10 GO terms (biological process, molecular function and cellular component) enriched by 33 DEGs discovered by at least 3 out of the 6 datasets with >40 samples using the new strategy proposed in this study (ordered by the number of genes enriched under each GO term).

| ID | GO Number | GO Term | Genes Enriched under the Corresponding GO Term | *p*-value |
|---|---|---|---|---|
| **Biological Process (BP)** | | | | |
| BP1 | GO:0048584 | Positive regulation of response to stimulus | ALB; ANKRD1; PLCG1; RAPGEF1; RGN; S100A8; TAC1; TNFSF10; TRAT1 | 6.13E-06 |
| BP2 | GO:0051049 | Regulation of transport | ANKRD1; KCNA3; PLCG1; RGN; S100A8; SCN1B; TAC1; TRAT1 | 3.20E-05 |
| BP3 | GO:0009605 | Response to external stimulus | ADM; ALB; ANKRD1; CP; HOXB9; S100A8; SCN1B; TAC1 | 3.42E-05 |
| BP4 | GO:0006955 | Immune response | ADM; KDM5D; MEFV; S100A8; TAC1; TNFSF10; TRAT1 | 1.09E-05 |
| BP5 | GO:0007267 | Cell-cell signaling | ADM; CRB1; FJX1; SCN1B; TAC1; TNFSF10 | 1.60E-05 |
| BP6 | GO:0009887 | Organ morphogenesis | ADM; ANKRD1; CRB1; FJX1; MGP; MICAL2 | 2.68E-05 |
| BP7 | GO:1902533 | Positive regulation of intracellular signals | ANKRD1; PLCG1; RAPGEF1; S100A8; TNFSF10; TRAT1 | 3.37E-05 |
| BP8 | GO:0050832 | Defense response to fungus | ADM; S100A8; TAC1 | 3.03E-06 |
| BP9 | GO:0009620 | Response to fungus | ADM; S100A8; TAC1 | 7.28E-06 |
| BP10 | GO:0002027 | Regulation of heart rate | ADM; SCN1B; TAC1 | 3.31E-05 |
| **Molecular Function (MF)** | | | | |
| MF1 | GO:0046914 | Transition metal ion binding | ALB; CP; KDM5D; MEFV; MICAL2; RGN; S100A8 | 5.12E-05 |
| MF2 | GO:0005102 | Receptor binding | ADM; PEX14; PLCG1; S100A8; TAC1; TNFSF10; TRAT1 | 7.13E-05 |
| MF3 | GO:0008270 | Zinc ion binding | ALB; KDM5D; MEFV; MICAL2; RGN; S100A8 | 1.54E-04 |
| MF4 | GO:0005509 | Calcium ion binding | CRB1; MGP; PLCG1; RGN; S100A8 | 1.32E-04 |
| MF5 | GO:0008092 | Cytoskeletal protein binding | ANKRD1; MEFV; MICAL2; PEX14; S100A8 | 2.79E-04 |

| | | | | |
|---|---|---|---|---|
| MF6 | GO:0048037 | Cofactor binding | ALB; CCBL2; MICAL2 | 8.90E-04 |
| MF7 | GO:0005504 | Fatty acid binding | ALB; S100A8 | 2.29E-04 |
| MF8 | GO:0030170 | Pyridoxal phosphate binding | ALB; CCBL2 | 6.48E-04 |
| MF9 | GO:0005507 | Copper ion binding | ALB; CP | 6.99E-04 |
| MF10 | GO:1990782 | Protein tyrosine kinase binding | PLCG1; TRAT1 | 6.99E-04 |
| **Cellular Component (CC)** | | | | |
| CC1 | GO:0005615 | Extracellular space | ADM; ALB; CP; FJX1; PCSK6; S100A8; TAC1; TNFSF10 | 4.51E-06 |
| CC2 | GO:0042995 | Cell projection | CRB1; KCNA3; MEFV; PLCG1; SCN1B; TAC1 | 1.53E-03 |
| CC3 | GO:0031226 | Intrinsic component of plasma membrane | CP; KCNA3; SCN1B; TNFSF10; TRAT1 | 6.08E-03 |
| CC4 | GO:0030424 | Axon | KCNA3; SCN1B; TAC1 | 3.33E-03 |
| CC5 | GO:0031012 | Extracellular matrix | ALB; MGP; PCSK6 | 3.51E-03 |
| CC6 | GO:0098797 | Plasma membrane protein complex | KCNA3; SCN1B; TRAT1 | 5.79E-03 |
| CC7 | GO:0072562 | Blood microparticle | ALB; CP | 4.77E-03 |
| CC8 | GO:0001726 | Ruffle | MEFV; PLCG1 | 5.64E-03 |
| CC9 | GO:0034703 | Cation channel complex | KCNA3; SCN1B | 6.44E-03 |
| CC10 | GO:0030027 | Lamellipodium | MEFV; PLCG1 | 6.81E-03 |

**Supplementary Table S9.** Transcription factor binding sites of the 33 DEGs identified in this study (ordered by the number of DEGs enriched in each binding site) confirmed by the published literatures.

| Transcription Factor (TF) Binding Sites | *p*-value | FDR *q*-value | No. of DEGs | The Relevance between SCZ and the TFs Identified in This Study |
|---|---|---|---|---|
| GGGAGGRR_V$MAZ_Q6 | 1.62E-04 | 4.99E-02 | 8 | MAZ was confirmed by the relative real time PCR as significantly down-regulated in SCZ patients[1], and was implicated as associated with cognitive disorder[2]. |
| RYTTCCTG_V$ETS2_B | 1.10E-04 | 4.99E-02 | 6 | The crucial but not exclusive contribution of the D21S17-ETS2 region to the cognitive impairments was confirmed by a nest design strategy[3,4]. |

**References for Table S9:**


1 N. A. Bowden; J. Weidenhofer; R. J. Scott; U. Schall; J. Todd; P. T. Michie; P. A. Tooney. Preliminary investigation of gene expression profiles in peripheral blood lymphocytes in schizophrenia. Schizophr Res. 2006, 82(2-3): 175-83

2 K. L. Jordan-Sciutto; J. M. Dragich; J. Caltagarone; D. J. Hall; R. Bowser. Fetal Alz-50 clone 1 (FAC1) protein interacts with the Myc-associated zinc finger protein (ZF87/MAZ) and alters its transcriptional activity. Biochemistry. 2000, 39(12): 3206-15

3 Z. Seregaza; P. L. Roubertoux; M. Jamon; B. Soumireu-Mourat. Mouse models of cognitive disorders in trisomy 21: a review. Behav Genet. 2006, 36(3): 387-404

4 P. Kahlem. Gene-dosage effect on chromosome 21 transcriptome in trisomy 21: implication in Down syndrome cognitive disorders. Behav Genet. 2006, 36(3): 416-28
